# Full-Length Transcriptome Analysis of Four Different Tissues of *Cephalotaxus oliveri*

**DOI:** 10.3390/ijms22020787

**Published:** 2021-01-14

**Authors:** Ziqing He, Yingjuan Su, Ting Wang

**Affiliations:** 1 School of Life Sciences, Sun Yat-sen University, Guangzhou 510275, China; hezq9@mail3.sysu.edu.cn; 2 Research Institute of Sun Yat-sen University in Shenzhen, Shenzhen 518057, China; 3 College of Life Sciences, South China Agricultural University, Guangzhou 510642, China

**Keywords:** *Cephalotaxus oliveri*, SMRT, transcriptome, multiple tissues, gene families, pathways

## Abstract

*Cephalotaxus oliveri* is a tertiary relict conifer endemic to China, regarded as a national second-level protected plant in China. This species has experienced severe changes in temperature and precipitation in the past millions of years, adapting well to harsh environments. In view of global climate change and its endangered conditions, it is crucial to study how it responds to changes in temperature and precipitation for its conservation work. In this study, single-molecule real-time (SMRT) sequencing and Illumina RNA sequencing were combined to generate the complete transcriptome of *C. oliveri*. Using the RNA-seq data to correct the SMRT sequencing data, the four tissues obtained 63,831 (root), 58,108 (stem), 33,013 (leaf) and 62,436 (male cone) full-length unigenes, with a N50 length of 2523, 3480, 3181, and 3267 bp, respectively. Additionally, 35,887, 11,306, 36,422, and 25,439 SSRs were detected for the male cone, leaf, root, and stem, respectively. The number of long non-coding RNAs predicted from the root was the largest (11,113), and the other tissues were 3408 (stem), 3193 (leaf), and 3107 (male cone), respectively. Functional annotation and enrichment analysis of tissue-specific expressed genes revealed the special roles in response to environmental stress and adaptability in the different four tissues. We also characterized the gene families and pathways related to abiotic factors. This work provides a comprehensive transcriptome resource for *C. oliveri*, and this resource will facilitate further studies on the functional genomics and adaptive evolution of *C. oliveri*.

## 1. Introduction

*Cephalotaxus oliveri,* a perennial shrub or small tree, belongs to the Genus *Cephalotaxus* sect. Pectinate in Cephalotaxaceae, and it is famous for producing the alkaloids harringtonine and homoharringtonine, which are effective in treating leucocythemia [1]. *C. oliveri* has been regarded as a vulnerable species by International Union for Conservation of Nature (IUCN) due to the deforestation and overexploitation [2,3]. Its natural populations were widely distributed, which can be found in the montane regions of northern Guangdong, Guizhou, western Hubei, Hunan, eastern Jiangxi, southern and western Sichuan, and eastern Yunnan in China [2]. The available paleobotanical data show that *C. oliveri* was widely distributed in North America, Europe, and Asia during the Cretaceous and Tertiary periods. After the Quaternary, due to the influence of glacier activities, *C. oliveri* only survived in a small environment. The existing *C. oliveri* is mainly distributed in the sandy shale mountains with an altitude of 1300–1500 m in eastern China, while in the western part it is distributed in the mountains with an altitude of 1500–2700 m, with the extreme lowest temperature reaching −5 °C [2,4]. As an old rare species with a long life, *C. oliveri* has experienced severe changes in temperature and precipitation over the past millions of years, adapting well to cold and arid environments [3]. In view of the continuous changes in the global climate, it is crucial to study how *C. oliveri* responds to the changes in temperature and precipitation for its conservation work. However, previous studies only use different molecular markers to study the genetic diversity, population history and adaptive loci of *C. oliveri* [3,5,6,7]. The molecular and genetic resources of this long-lived conifer are still blank, limiting the studies on molecular and genetic analysis.

To adapt to changes in the environment, conifers have evolved a variety of physiological responses and defense systems for withstanding various stress conditions, most of which depend on changes in gene expression [8,9]. Researchers can study the expression of all genes in specific tissues or cells at the overall level based on transcriptome studies, from which they can mine gene families and molecular pathways related to environmental stress or adaptation. Several important candidate genes and metabolic pathways related to abiotic stress have been characterized in some conifer species. For example, Meng et al. found that MAPK signaling pathway and TF families, like NAC, WRKY, ERF, MYB, and bZIP responded to cold stress in *Taxus chinensis* based on transcriptomic analyses [10]. Moreover, Fox et al. created a drought-stressed *Pinus halepensis* transcriptome and found the accumulation of heat shock proteins, thaumatin and exordium and the genes related to abscisic acid response were up-regulated [11]. Other gene products, like dehydrins, were also found to be related to environmental stress in some conifers [12,13,14]. In order to have a more comprehensive understanding of the transcriptome information of *C. oliveri* and its genes and metabolic pathways related to cold and drought tolerance, it is necessary to conduct transcriptome studies on multiple tissues of *C. oliveri*.

The development of high-throughput sequencing technology has made transcriptome sequencing widely used, greatly improving the efficiency of gene discovery [15,16,17,18]. The development of sequencing technology has provided scientists with powerful tools for studying plant transcriptomes. Illumina sequencing technology and third-generation single molecular real-time (SMRT) sequencing technology have their own advantages. The former has the advantages of high throughput, high sensitivity, and high accuracy; and its cost is lower than that of third-generation sequencing; but its read length is short, and a complex splicing process is required after sequencing [19]. The latter has the advantages of ultra-long sequence reading, no need for PCR amplification and direct detection of DNA modifications, but its sequencing accuracy is lower than that of second-generation sequencing, only 85% [20,21]. Therefore, combining second-generation and third-generation sequencing technologies is commonly used in transcriptomic analyses.

Here, we combined long read SMRT sequencing and short read RNA-Seq to analyze the transcriptome of *C. oliveri*. The aims of our study include: (i) generating reference transcriptome sequences and annotating the transcriptome for the four tissues of *C. oliveri*; (ii) identifying transcription factors (TFs), long non-coding RNAs (lncRNAs), and simple sequence repeats (SSRs); (iii) exploring gene expression patterns among the four organs; (iv) exploring the functions of tissue-specific expressed unigenes; and (v) charactering the candidate gene families and metabolic pathways related to biotic and abiotic stress. This is the first report of a comprehensive characterization of the global transcriptome of *C. oliveri*. This study provides a valuable genetic resource for further studies on functional genomic and adaptive evolution of this species and related species.

## 2. Results

### 2.1. The Full-Length Sequences of Pacbio Iso-Seq

Based on SMRT sequencing technology, 560,605, 312,574, 353,127 and 213,338 polymerase reads were generated for the male cone, root, stem, and leaf (Supplementary Materials Table S1). After preprocessing, 9,465,158, 911,365, 8,444,499, 7,873,312 subreads were obtained for the male cone, stem, root, and leaf with a mean length of 2127, 2097, 1238, 1912 bp, respectively. Then subreads were used to self-correction to obtain circular consensus sequences (CCSs, Figure S1). In total, 264,227, 303,178, 189,764 and 462,122 CCSs were obtained for the root, stem, leaf, and male cone, respectively. In addition, 213,507, 268,740, 150,247, and 401,815 sequences were identified as full-length non-chimeric (FLNC) reads from the CCSs for the root, stem, leaf, and male cone. Based on the iterative clustering for error correction (ICE) algorithm and the polishing using the Arrow algorithm, 143,177, 158,855, 96,459 and 209,217 high-quality, full-length, and polished consensus isoforms were generated for the root, stem, leaf, and male cone, respectively. After error correction using the RNA-Seq data and removing the redundant sequences using CD-HIT, 63,931, 58,108, 33,013, and 62,436 unigenes were obtained for the root, stem, leaf, and male cone, with the N50 of 2523 bp, 3480 bp, 3181 bp and 3267 bp, respectively (Table S2). Based on benchmarking universal single-copy ortholog (BUSCO) analyses, approximately 62.7% (root), 72.7% (male cone), 64.6% (leaf) and 70.5% (stem) of the 1440 expected embryophyte genes were identified as complete (Figure S2).

### 2.2. De Novo Assembly of Illumina RNA-Seq Data

The Illumina RNA-seq generated 210.48 million raw reads for all tissue samples. After trimming and filtering, 52.58, 50.07, 48.63, and 53.48 million clean reads were obtained for the root, stem, leaf, and male cone (Table S3). Each sample produced no less than 7 Gb data, the percentage of Q30 reached more than 91.94%, and the GC content was between 43.95% and 44.88%. Based on these clean reads, Trinity software de novo assembled 72,356, 43,372, 42,844 and 36,446 unigenes for the root, stem, male cone, and leaf. The corresponding N50 was 1905 bp, 2150 bp, 2051 bp and 2046 bp, respectively (Table S4), which were shorter than the N50 length from PacBio isoform sequencing (Pacbio Iso-Seq).

### 2.3. Functional Annotation

To obtain a comprehensive annotation of *C. oliveri* transcriptome, 62,436 (male cone), 58,108 (stem), 33,013 (leaf), 63,831 (root) full-length transcripts were annotated by searching against seven databases, including NCBI non-redundant protein (Nr), NCBI non-redundant nucleotide (Nt), Swiss-Prot, Kyoto Encyclopedia of Genes and Genomes (KEGG), Gene Ontology (GO), Pfam protein families, and NCBI euKaryotic Ortholog Groups (KOG) databases. Among the four tissues, male cone was the best annotated, followed by stem, leaf, and root. 58,601 (93.86%) unigenes of male cone were annotated in at least one database, while the number of the other three tissues was 58,108 (91.19%, stem), 29,411 (89.09%, leaf), and 51,054 (79.98%. root), respectively (Table 1).

The homologous species of *C. oliveri* were predicted by sequence alignment on the basis of the Nr database. The species distribution of the top 20 best match results in Nr were shown in Figure S3. The top three homologous species for the four tissues were *Picea sitchensis*, *Amborella trichopoda* and *Nelumbo nucifera*. The number of unigenes distributed in these three species was 17,393 (30.10%), 10,676 (18.47%) and 5530 (9.57%) for male cone; 10,304 (35.97%), 4098 (14.30%) and 2202 (7.69 %) for leaf; 12,774 (26.40%), 4227 (8.74%) and 3099 (6.22%) for root; 14,459 (28.01%), 8245 (15.97%) and 4544 (8.80%) for stem. As expected, the top homologous species was a conifer.

GO terms were assigned to *C. oliveri* unigenes for functional categorization. The assigned GO terms were summarized into the three main GO categories (biological process, cellular component, and molecular function), and then into 54 functional categories (Figure S4). Within the biological process category, “metabolic process” (GO: 0008152), “cellular process” (GO: 0009987) and “single-organism process” (GO: 0044699) were prominent subcategories. For the cell components category, the first three subcategories of the four tissues were “cell” (GO: 0005488), “cell part” (GO: 0044464), and “organelle” (GO: 0043226). In the molecular function category, the first three subcategories of the four tissues were the same as “binding” (GO: 0005488), “catalytic activity” (GO: 0003824) and “transporter activity” (GO: 0005215) (Figure S2). In addition, there were 3555 (male cone), 1746 (leaf), 2878 (root) and 3332 (stem) unigenes annotated into the “response to stimulus” (GO: 0050896) in the biological process category. In the next level of GO terms of “response to stimulus”, that is, the third level of GO terms, there are five GO terms worthy of attention. *C. oliveri* had 218 (male cone), 124 (leaf), 207 (root) and 190 (stem) unigenes annotated to “response to stress” (GO: 0006950). There were 78 (male cone), 24 (leaf), 39 (root) and 68 (stem) unigenes assigned to “response to hormone stimulus” (GO: 0009725). The number of unigenes annotated to “response to water” (GO: 0009415) were 28 (male cone), 53 (leaf), 43 (root) and 63 (stem), respectively. It was worth noting that the number of unigenes annotated to “response to oxidative stress” (GO: 0006979) in the root tissue was 105, which was significantly higher than the other three tissues (Table 2).

It is helpful to evaluate the biological pathways that might be active in *C. oliveri* with KEGG pathway analysis. According to the KEGG classification, the unigenes were assigned to six KEGG biochemical pathways: (1) cellular processes, (2) environmental information processing, (3) genetic information processing, (4) metabolism, (5) organismal systems and (6) human diseases (Figure S5). The top five subcategories of the four tissues were “transport and catabolism”, “folding”, “sorting and degradation”, “translation”, “signal transduction”, and “carbohydrate metabolism”. Obviously, the category of “environment information processing” was worthy of our attention. Among the four tissues, 2893 (male cone), 1409 (leaf), 2503 (root) and 2695 (stem) unigenes were assigned to “environment information processing”. This category is mainly divided into three subcategories, namely “membrane transport”, “signal transduction” and “signaling molecules and interaction”. In addition, the sub-category of “environmental adaptation” under “organismal systems” also deserves special attention. 586 (male cone), 326 (leaf), 548 (root) and 613 (stem) unigenes were assigned to this category, the specific ko pathways were plant–pathogen interaction (ko04626) and circadian rhythm-plant (ko04712).

**Figure 1:**
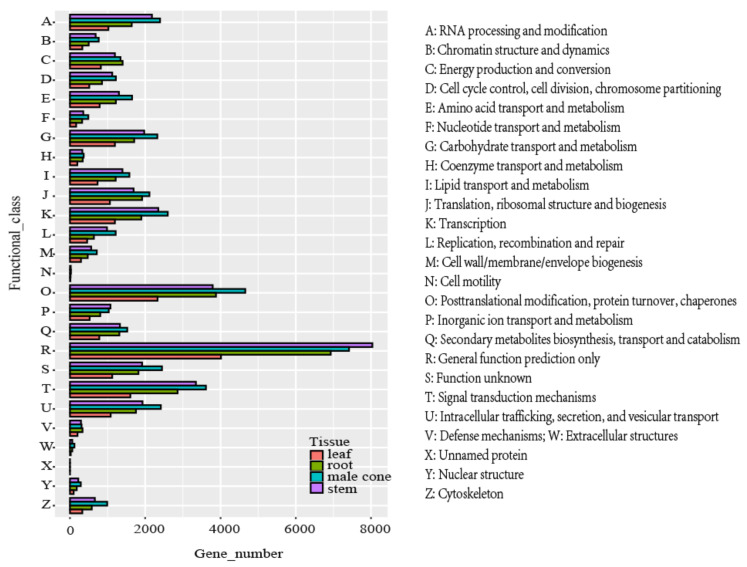
Eukaryotic ortholog group (KOG) classification of unigenes.

The KOG analysis demonstrated that 38,768 (male cone), 34,613 (stem), 18,671 (leaf), 31,056 (root) unigenes were assigned to 26 functional clusters (Figure 1). In the four tissues, the first five categories were “general function prediction (R)”, “post-translational modification, protein conversion, molecular chaperone (O)”, “signal transduction mechanism (T)”, “transcription (K)” and “unknown function (S)”. In addition, 1519 (male cone), 778 (leaf), 1310 (root) and 1326 (stem) unigenes were assigned to the “biosynthesis, transport, and catabolism of secondary metabolites (Q)”; 305 (male cone), 200 (leaf), 332 (root) and 294 (stem) unigenes were annotated to “defense mechanism (V)”. In the Q category, most of the unigenes were annotated as cytochrome P450 CYP4/CYP19/CYP26 subfamilies (KOG0157), iron/ascorbate family oxidoreductases (KOG0143), pleiotropic drug resistance proteins (PDR1-15) (KOG0065), cytochrome P450 CYP2 subfamily (KOG0156) and multidrug/pheromone exporter (KOG0055).

### 2.4. Identification of TFs, SSRs and LncRNAs

**Figure 2:**
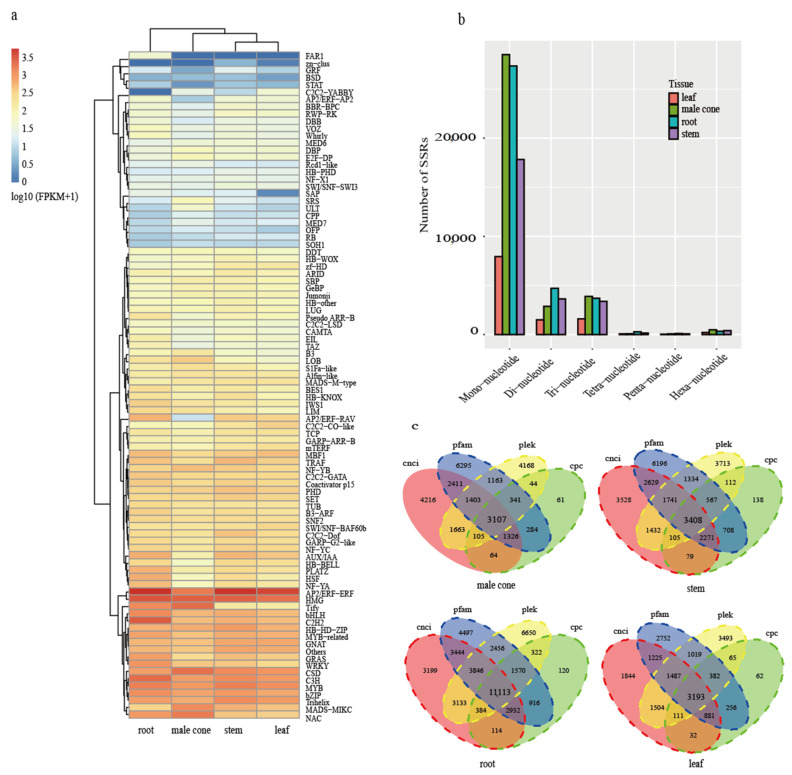
Identification of the transcription factors (TFs), simple sequence repeats (SSRs) andlong non-coding RNAs (lncRNAs). (**a**) Expression profiles of transcription factor families in four tissues; (**b**) the histogram representing the number of different types of SSRs; (**c**) Venn diagram of lncRNAs predicted by four different methods.

Transcription factor (TF) can specifically bind to a specific sequence upstream of the 5’ end of a gene, thereby regulating gene expression patterns. In this study, iTAK pipeline was used to identify plant transcription factors in four tissues of *C. oliveri*. A total of 3846 (male cone), 1662 (leaf), 2522 (root) and 3685 (stem) transcripts were predicted as plant transcription factors (Table S5). Except for FAR1, zn-clus, and C2C2-YABBY, all other transcription factor families had a certain amount of expression in the four tissues of *C. oliveri* (Figure 2a). The top 20 transcription factor families with the highest expression in *C. oliveri* were AP2/ERF−ERF, HMG, CSD, C3H, MYB, C2H2, Trihelix, bZIP, Tify, NAC, bHLH, HB−HD−ZIP, MADS −MIKC, MYB−related, GNAT, WRKY, GRAS, AUX/IAA, MBF1 and TRAF.

SSR is widely and evenly distributed in the genome of eukaryotes. The software MISA was used to search the SSR profiles in the unigenes of *C. oliveri*. 20,911 (33.49%), 8239 (24.96%), 20,857 (32.68%) and 16,148 (27.79%) SSR-containing unigenes were detected from male cone, leaf, root, and stem, respectively. In the four tissues, mono-nucleotide repeat motifs were the most abundant, followed by di-nucleotide repeats and tri-nucleotide repeats (Figure 2b).

Four computational approaches (CPC, CNCI, CPAT, and Pfam) were combined to identify lncRNAs. As is shown in Figure 2c, 11,113 (root), 3408 (stem), 3193 (leaf), and 3107 (male cone) unigenes were predicted as lncRNAs by the four methods, accounting for 5.50%, 6.63%, 2.41%, and 4.57% of the total unigenes, respectively.

### 2.5. Gene Expression Level Analysis

**Figure 3:**
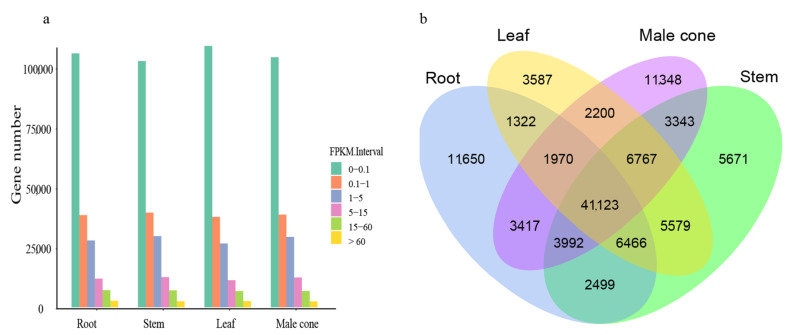
Analysis of gene expression in four tissues of *C. oliveri*. (**a**) The fragments per kilobase of transcript sequence per million base pairs sequenced (FPKM) interval distribution in four tissues; (**b**) A Venn diagram of the number of unigenes expressed in four tissues.

To investigate the expression patterns of unigenes in *C. oliveri*, the Illumina clean reads were mapped to the SMRT non-redundant transcripts to determine expression level using fragments per kilobase of transcript sequence per million base pairs sequenced (FPKM). The mapped reads of each tissue were 48,227,272 (90.18%, male cone), 43,922,320 (90.31%, leaf), 33,697,206 (64.08%, root) and 44,716,176 (89.30%, stem), respectively (Table S6). In the four tissues, the number of unigenes in different expression intervals was almost the same. The four tissues had 2656 (1.37%, root), 2436 (1.26%, stem), 2503 (1.29%, leaf) and 2397 (1.24%, male cone) unigenes with high expression, and their expression levels were greater than 60 FPKM (Figure 3a). Using FPKM > 0.3 as the standard for unigenes expression, a Venn diagram of unigene expression in four tissues was drawn and a total of 41,123 unigenes expressed in all tissues were found (Figure 3b). In addition, the number of genes uniquely expressed in each tissue was 11,650 (root), 3587 (leaf), 11,348 (male cone) and 5671 (stem).

Pairwise comparisons among the four tissues were performed to identify the differentially expressed unigenes (DEGs). The largest differences were observed between male cone and root, in which 12,021 DEGs were detected. The smallest difference existed between root and stem, in which only 1634 DEGs were detected. In the male cone versus leaf, male cone versus stem, leaf versus root and root versus stem, 10,343, 9465, 9704 and 8385 differentially expressed unigenes were identified, respectively (Table 3).

### 2.6. GO Enrichment of Tissue-Specific Expressed Genes

In order to explore the functions of these tissue-specific expressed unigenes, GO functional enrichment analysis was conducted separately for the four tissues. GO enrichment analyses showed that tissue-specific expressed unigenes were enriched in biological processes and molecular function varying across tissues (Table S7).

**Figure 4:**
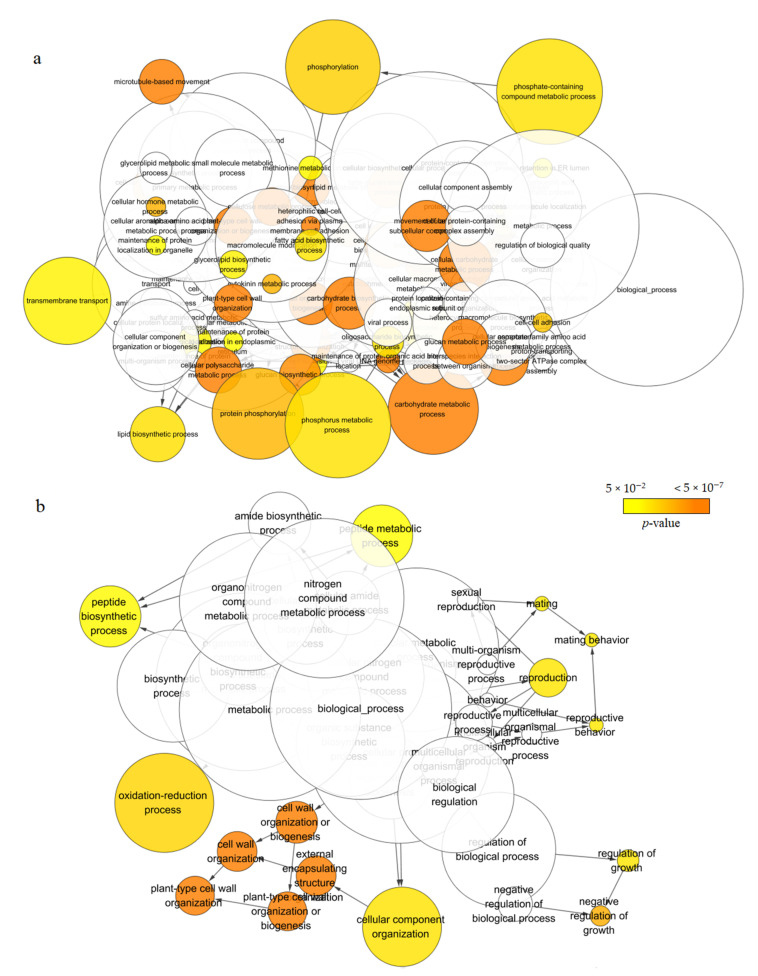
The “biological process” category of Gene Ontology (GO) enrichment analysis of tissue-specific expressed genes. (**a**) male cone; (**b**) root.

In the “biological process” category, the four tissues were enriched to 51 (male cone), 15 (root), 9 (leaf) and 5 (stem) GO terms (Figure 4 and Figure S6), respectively. The first five GO terms for male cone were “phosphate-containing compound metabolic process” (GO: 0006796, 705 unigenes), “phosphorus metabolic process” (GO: 0006793, 705), “phosphorylation” (GO: 0016310, 555), “protein phosphorylation” (GO: 0006468, 516), “carbohydrate metabolic process” (GO: 005975, 500) (Figure 4a). This indicated that phosphorylation may play a very important role in male cone. The tissue-specific expressed genes in stem were mainly assigned to five regulatory processes, namely “regulation of protein metabolic process” (GO: 0051246, 79), “regulation of cellular protein metabolic process” (GO: 0032268, 76), “posttranscriptional regulation of gene expression” (GO: 0010608, 76), “regulation of translation” (GO: 0006417, 74) and “regulation of cellular amide metabolic process” (GO: 0034248, 74) (Figure S6a). The tissue-specific expressed genes in leaf were enriched in three GO terms related to response to biological stress, namely “response to defenses of other organisms” (GO: 0052173), “response to host defenses” (GO: 0052200) and “response to host” (GO: 0075136) (Figure S6b).

In the “molecular function” category, the specifically expressed genes of male cone were significantly enriched in more categories than the other tissues, and were mainly enriched in various active molecules and binding molecules (Figures S7 and S8). Although root-specific expressed genes are also enriched in active molecules, its categories were far less than those in male cone. In stem and leaf tissues, specifically expressed genes were mainly enriched in various binding molecules.

### 2.7. KEGG Enrichment of Tissue-Specific Expressed Unigenes

**Figure 5:**
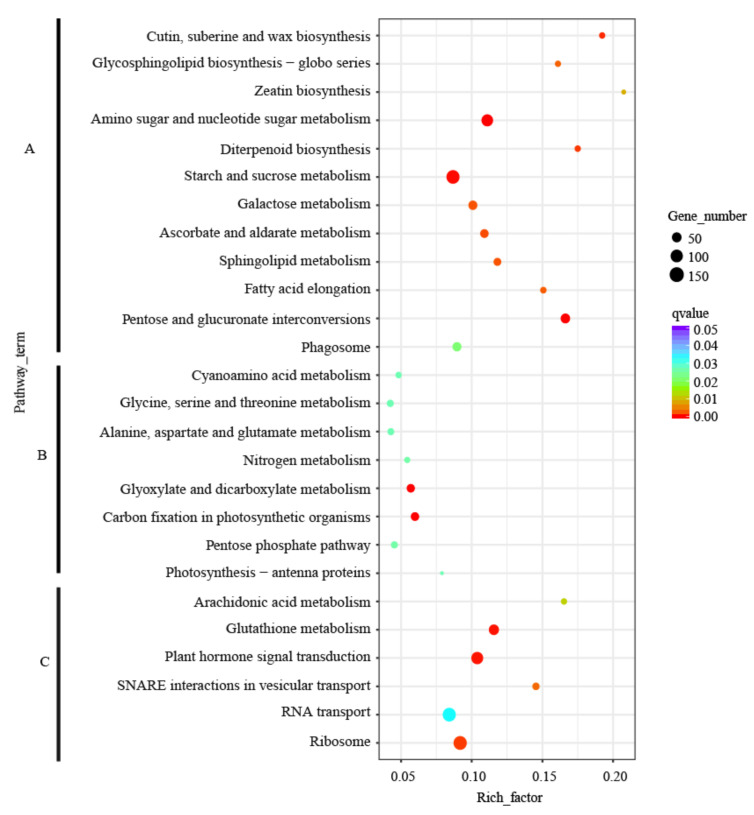
The Kyoto Encyclopedia of Genes and Genomes (KEGG) pathway enrichment analysis of tissue-specific expressed genes (**A**): male cone; (**B**): leaf; (**C**): root.

The significant enrichment of different biological pathways can help to identify metabolic and signal transduction pathways that tissue-specific expressed unigenes were involved in. A KEGG enrichment analysis of all tissue-specific expressed unigenes was conducted and found that only the stem-specific unigenes were not significantly enriched in any KEGG pathway, and the pathways enriched in the other three tissues were different (Table S8, Figure 5). It was noteworthy that the male cone-specific expressed unigenes were significantly enriched in four biosynthetic pathways, namely “diterpenoid biosynthesis” (ko00904, 21), “cutin, suberine and wax biosynthesis” (ko00073, 20), “glycosphingolipid biosynthesis-globo series” (ko00603, 19) and “zeatin biosynthesis” (ko00908, 11). In leaf, the tissue-specific expressed unigenes were enriched in amino acid metabolism pathways, including “glycine, serine and threonine metabolism” (ko00260, 29), “alanine, aspartate and glutamate metabolism” (ko00250, 27) and “cyanoamino acid metabolism” (ko00460, 21). In addition, the tissue-specific expressed unigenes from leaf were significantly enriched in the pathways related to photosynthesis, such as “carbon fixation in photosynthetic organisms” (ko00710, 50) and “photosynthesis-antenna proteins” (ko00196, 9), which were in line with expectations.

### 2.8. Gene Families

**Figure 6:**
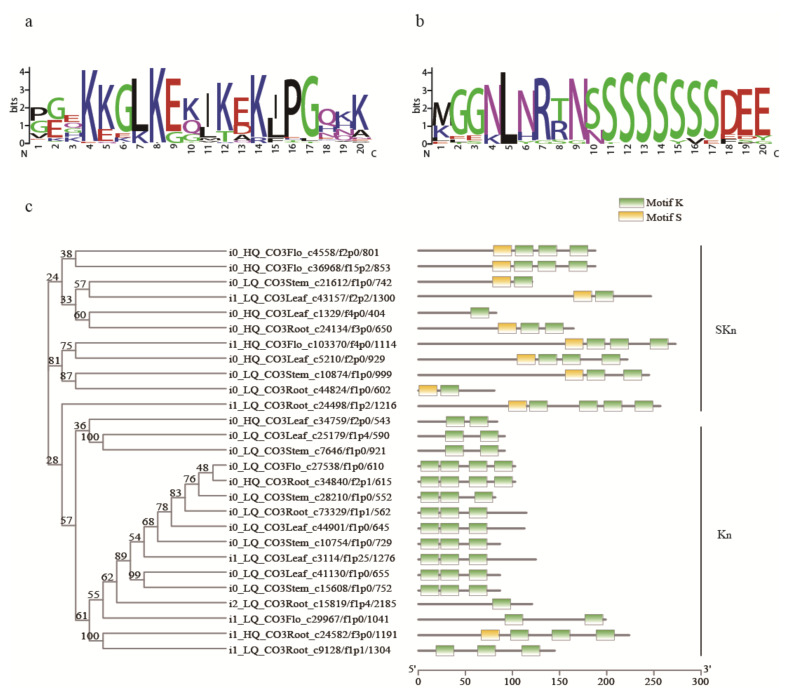
Conserved motif and neighbor-joining (NJ) phylogenetic tree of dehydrins. (**a**) K motif; (**b**) S motif; (**c**) NJ tree.

**Figure 7:**
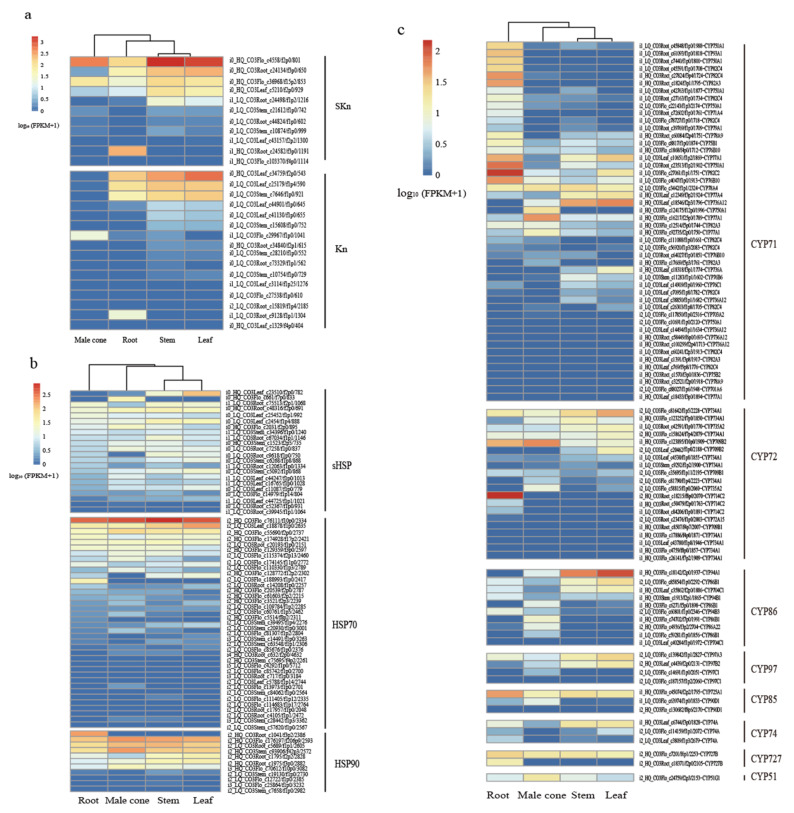
Expression profiles of different genes families in four tissues. (**a**) dehydrin (DHN) genes families; (**b**) heat shock protein (HSP) genes families; (**c**) cytochrome P450 (CYP450) genes families.

A total of 84 complete sequences annotated as dehydrin were mined from the full-length transcriptome data (Table S9). After deleting sequences that were too short (<80 aa) and too long (>600 aa), there were 67 sequences remaining in the four tissues. CD-HIT was used to perform a de-redundancy analysis on 67 sequences with 100% similarity, removing the identical sequences in the four tissues, leaving a total of 32 sequences. The MEME program in MEME Suite 5.1.0 was used to conduct an exhaustive search on the conservative amino acid motifs of the 32 sequences. Ten motifs were identified from the dehydrins of *C. oliveri*, from which lysine-rich K motif (Figure 6a) and serine-rich S motif (Figure 6b) were found, and did not find E motif, A motif and Y motif. The neighbor-joining phylogenetic tree (NJ tree) showed that all sequences were divided into three groups (Figure 6c). It was found that most of the sequences with K_n_ structures were grouped into one category, and the sequences with the SK_n_ structure were divided into two groups due to the position of the motifs. It can be seen from the expression heat map that K_n_ and SK_n_ type dehydrins were mainly expressed in the root, stem, and leaf, but their expression level in stem and leaf were significantly higher than the other two tissues (Figure 7a). In addition, only SK_n_ type dehydrins were expressed in the male cone with a low level.

A total of 181 complete sequences annotated as heat shock proteins were identified from the full-length transcriptome data (Table S10). Use ExPASy tool (http://www.expasy.org/tools/) to predict the theoretical isoelectric point (PI) and molecular weight (kDa) of the identified heat shock proteins. Among the 211 sequences, 85 had molecular weights ranging from 5 to 43 kDa, 27 were from 45 to 65 kDa, 53 were from 66 to 78 kDa, 40 were from 83 to 97 kDa, and 6 were from 100 to 102 kDa. From the annotation results of Nr and Swissprot, it was found that these heat shock proteins were only annotated as the small molecule heat shock protein (sHSP), HSP70 and HSP90 families. Therefore, combining the predicted molecular weight and annotation results, a total of 22 sequences were identified as the sHSP family, 38 sequences were identified as the HSP70 family, and 11 sequences were identified as the HSP90 family.

It can be seen from the expression heat map that most sHSP protein families had certain expression levels in the four tissues of *C. oliveri* (Figure 7b). Although there were many sequences annotated as HSP70 protein family, only 12 sequences were obviously highly expressed in the four tissues. In the HSP90 protein family, 3 sequences were highly expressed in the four tissues. In addition, the expression level of i2_HQ_CO3Root_c1041/f3p2/2386 in the root was significantly higher than that of the other three tissues.

**Figure 8:**
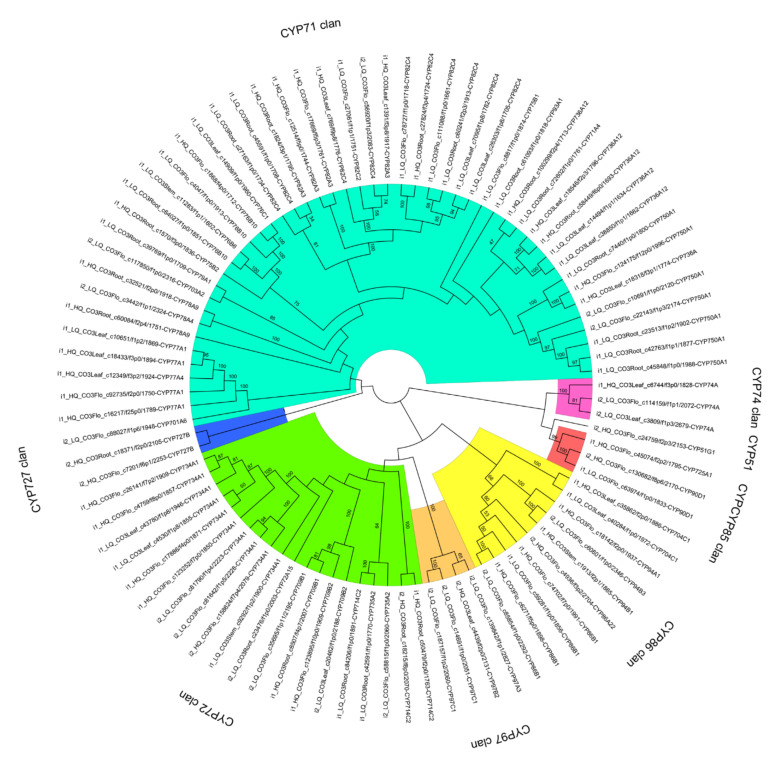
Phylogenetic analyses performed with 256 cytochrome P450 (CYP450) sequences of *C. oliveri*.

A total of 493 complete sequences annotated as cytochrome CYP were found in the four tissues of *C. oliveri*, of which 195 transcripts were >500 aa (Table S11). 195 cytochrome CYP enzyme sequences were clustered with 95% similarity to eliminate redundancy, and were divided into 118 clusters. The representative sequences in 118 clusters were used as input, and local blastp alignment was performed with 12,112 cytochrome CYP enzyme sequences in the Nelson database to further confirm the sequence annotation results. Sequences with a homology of less than 40% were deleted, and 91 sequences remained, and MEGA-X was used to construct an NJ tree. The results show that different cytochrome CYP enzyme families are grouped together (Figure 8). All sequences of the four tissues can be divided into 8 clans: CYP71, CYP72, CYP86, CYP97, CYP85, CYP51, CYP74 and CYP727.

It can be seen from the expression heat map that most family members of the CYP71 clan were expressed in the root of *C. oliveri*, only some of the family members were expressed in the male cone, stem, and leaf (Figure 7c). On the other hand, members of the CYP72, CYP86, and CYP97 families were mainly expressed in stem and leaf. The expression levels of the rest of the family members were almost the same in the four tissues of *C. oliveri*.

### 2.9. Pathway Related to Environmental Adaptation

From the results of KEGG annotation, it can be found that there were certain KEGG pathways related to environmental stress in the four tissues. The gene products and tissue expression of genes involved in these pathways were further analyzed.

**Figure 9:**
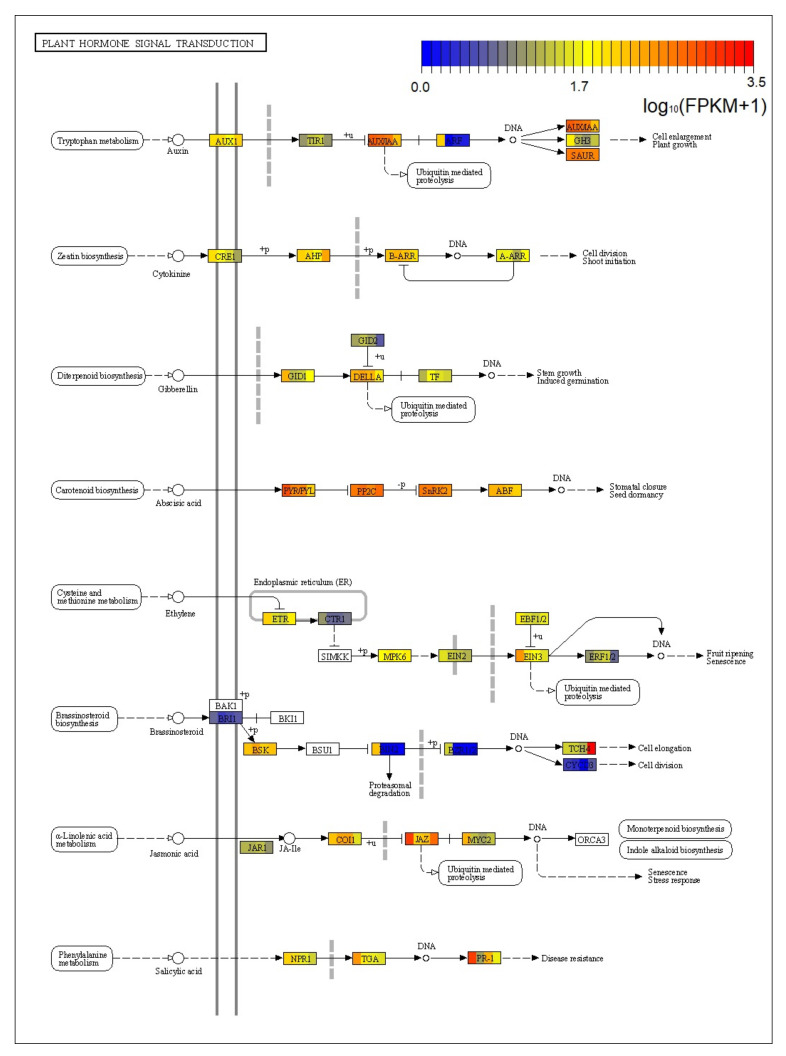
Metabolic pathway of plant hormone signal transduction in *C. oliveri.* The color boxes represent substances assigned at least one unigene in *C. oliveri*; the boxes are divided into four colors, representing the genes expression in the root, stem, leaf, and male cone in turn.

A total of 1299 unigenes from the four tissues were assigned to the Plant hormone signal transduction (ko04075) pathway (Table S12, Figure 9). This pathway mainly involves 40 different substances, which were mainly involved in the signal transduction of cytokinin, gibberellin, abscisic acid, ethylene, brassinosteroids, jasmonic acid and salicylic acid.

Most proteins encoded by unigenes were expressed in the four tissue (Figure 9). Moreover, JAZ (jasmonate ZIM domain-containing protein, K13464), IAA (auxin-responsive protein IAA, K14484), SAUR (SAUR family protein, K14488), PYL (abscisic acid receptor PYR/PYL family, K14496), PP2C (protein phosphatase 2C, K14497) and SNRK2 (serine/threonine-protein kinase, K14498) had high expression levels in four tissues. In addition, the expression of TCH4 (xyloglucan: xyloglucosyl transferase, K14504) in male cone was significantly higher than that of the other three tissues. The expression of ARF (auxin response factor, K14486), BIN2 (protein brassinosteroid insensitive 2, K14502) and BZR1_2 (brassinosteroid resistant 1/2, K14503) in the root were significantly higher than that of the other three tissues.

**Figure 10:**
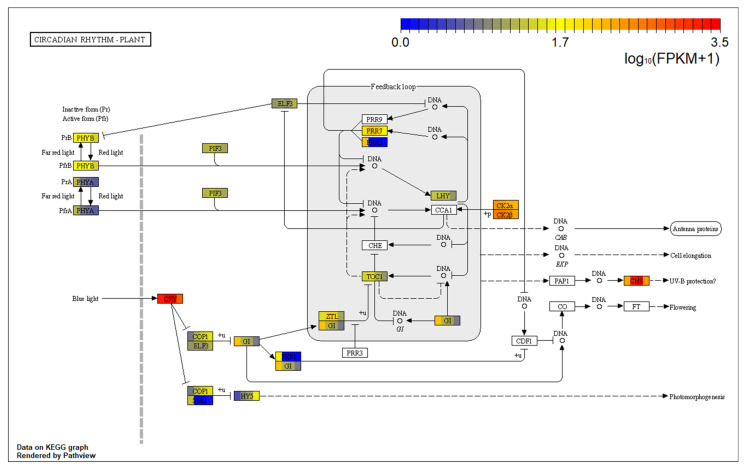
Metabolic pathway of circadian rhythm plant in *C. oliveri.* The color boxes represent substances assigned at least one unigene in *C. oliveri*; the boxes are divided into four colors, representing the genes expression in the root, stem, leaf, and male cone in turn.

A total of 495 unigenes from the four tissues were assigned to the circadian rhythm-plant (ko04712) pathway, involving a total of 19 substances (Table S13, Figure 10). The core oscillator of plant circadian rhythm is composed of core circuit, day circuit and night circuit. A total of 34, 12 unigenes coding for LHY and TOC1 of the core circuits were found in the four tissues, and 8, 80, 35, 20, and 15 unigenes were respectively annotated as PRR5, PRR7, GI, ZTL, which were the component for the daytime circuit or evening circuit. In addition, three families (CSNK2B, casein kinase II subunit beta, K03115; CSNK2A, casein kinase II subunit alpha, K03097; CHS, chalcone synthase, K00660) were highly expressed in all four tissues (Figure 10). The number of unigenes annotated for these three proteins varied from 22 to 66. It is worth noting that the genes encoding SPA1 (protein suppressor of PHYA-105 1, K16240), PRR5 (pseudo-response regulator 5, K12130) and FKF1 (flavin-binding kelch repeat F-box protein 1, K12116) only expressed in the root.

## 3. Discussion

### 3.1. Transcriptome Sequencing

This study combined the PacBio SMRT-Seq and Illumina RNA-Seq to analyze the full-length transcripts and genes expression patterns of the four tissues of *C. oliveri*. Based on Illumina sequencing, 72,356 (root), 43,372 (stem), 42,844 (male cone) and 36,446 (leaf) unigenes were obtained from the four tissues of *C. oliveri*, with N50 ranging from 1905 to 2061 bp. Based on SMRT sequencing technology, the number of unigenes in the four tissues was 63,831 (root), 58,108 (stem), 33,013 (leaf) and 62,436 (male cone), and the N50 range varied from 2523 bp to 3267 bp, which is significantly longer than the second-generation sequencing data. In addition, the BUSCO software was used to evaluate the completeness of the three-generation full-length transcripts of *C. oliveri*. 902 (root, 62.7%), 1048 (male cone, 72.7%), 930 (leaf, 64.6%) and 1015 (stem, 70.5%) complete core conserved genes were retrieved in the four tissues, which indicated the results of Iso-seq were good.

### 3.2. Funtional Annotation

More than 80% of unigenes in the four tissues of *C. oliveri* have been annotated in at least one database, which provides important information for studying the interaction mechanism between *C. oliveri* and the environment.

In the KOG classification, 1519 (male cone), 778 (leaf), 1310 (root) and 1326 (stem) genes were annotated as the “biosynthetic transport and catabolism” (Q). And 305 (male cone), 200 (leaf), 332 (root) and 294 (stem) unigenes were assigned to the “defense mechanism” (V). Secondary metabolites were non-nutritive substances in plants, so they were once considered as wastes during plant metabolism. However, after in-depth research on it, researchers found that secondary metabolites play an important role in plant growth and development and in responding to various biotic and abiotic stresses [22,23]. It is the long-term evolution of plants in the process of adapting to the living environment.

In the GO classification, 3555 (male cone), 1746 (leaf), 2878 (root) and 3332 unigenes were assigned to “response to stimulus” (GO: 0050896). In-depth analysis of this category, 239 (male cone), 163 (leaf), 234 (root) and 229 (stem) unigenes annotated as “response to stress” (GO: 0006950), 32 (male cone flowers), 64 (leaf), 50 (root) and 74 (stem) unigenes annotated as “response to water” (GO: 0009415) were found. These findings help us to further study the adaptability of *C. oliveri* in different environments. In addition, the number of unigenes assigned to “response to biotic stimulus” (GO: 0009607) in the root was significantly higher than that of the other three tissues. This indicated that the root, as an underground tissue, may require more genes and related substances to deal with biotic stress.

In the KEGG pathway analysis, four tissues had multiple unigenes assigned to “environmental information processing” and “environmental adaptation” categories. The sub-categories of “environmental information processing” mainly included the ABC transporters pathway (ko02010), the bacterial secretion system pathway (ko03070), AMPK signaling pathway (ko04152), calcium signaling pathway (ko04020), cAMP signaling pathway (ko04024) and MAPK signaling pathway-plant (ko04016). The sensing and transduction of stress signals are essential for plant adaptation and survival [24]. Ca^2+^ is a characteristic signal that responds to various abiotic stresses. Low temperature or drought stress will cause an increase in the concentration of Ca^2+^ in plant cells. This signal can be transmitted downstream through the calcium signal transduction pathway to activate the expression of corresponding resistance genes [24]. Liu Xueqin studied the signal transduction mechanism of calcium in the process of low temperature stress on *Cephalotaxus fortunei*, and found that Ca^2+^ treatment enhanced the activity of protective enzymes in the cell [25]. In addition, Ca^2+^ combined with CaM protein to transmit signals, activating the downstream target protein, and finally enhanced the cold resistance of *C. fortunei* [25]. MAPK signaling pathway is one of the most studied signal transduction mechanisms in plants. It is composed of a class of proteins that can amplify signals step by step through tertiary phosphorylation [26]. It has been reported that the MAPK signaling pathway is an important signaling pathway that regulates the cold response genes in *T. chinensis* [10].

In the category of environmental adaptation, it mainly involved plant–pathogen interaction pathway (ko04626), circadian rhythm-plant pathway (ko04712) and circadian rhythm pathway (ko04710). Circadian rhythm refers to the changes in life activities in a certain period of time, which participates in the synthesis of plant hormones, signal transduction and controls the stability of plant nutrients and the concentration of certain osmotic adjustment substances [27]. Studies have shown that the CBF cold response pathway of *Arabidopsis thaliana* is simultaneously induced by low temperature and controlled by the biological clock [28]. The freezing tolerance of *Pinus sylvestris* was not only tissue specific, but also affected by the photoperiod and temperature of the timing factors of the biological clock [29]. In this study, there were more than 100 unigenes assigned to the circadian rhythm-plant (ko04712) in the four tissues. This group of unigenes was also worthy of further study.

### 3.3. Potential Aoles of Transcription Factors, LncRNAs and SSRs

Transcription factors were very important regulatory factors in plants. They play an important role in regulating the response of plants to various biotic and abiotic stresses, and activating downstream target genes to improve plant resistance [30]. In this study, 92 transcription factor families were identified in the four tissues of *C. oliveri*. Almost all transcription factor families were expressed in four tissues. The top 20 transcription factor families with the highest expression in the four tissues were AP2/ERF, ERF, HMG, Tify bHLH, C2H2, HB-HD-ZIP, MYB-related, GNAT, GRAS, WRKY, CSD, C3H, MYB, bZIP, Trihelix, MADS, MIKC, NAC, PLATZ, HSF, and NF-YA.

NAC plays an important role in plant development and response to environmental stimuli. Through RT-PCR experiments, Zhang found that *PwNAC2* in *Picea wilsonii* can be induced by a variety of abiotic stress and plant hormones, and can enhance plant tolerance to abiotic stress through ABA signal or other signal transduction pathways [31]. In the natural state, the FPKM of NAC in the four tissues were 1000.32 (root), 445.23 (stem), 371.17 (leaf) and 1562.61 (male cone), which was expected to play an important regulatory role in the environmental adaptability of *C. oliveri*. Zinc finger proteins were one of the largest TF families in plants [32]. According to its conserved domains, zinc finger proteins can be divided into C2H2, C3H, C3HC4, C2HC5, C4HC3, C2HC, C4, C6 and C8 subfamily. Only the C2H2 and C3H subfamily were annotated in the four tissues of *C. oliveri*. Studies have shown that C2H2 zinc finger protein can directly regulate the downstream genes related to cold in plants, increase the level of osmotic substances, and regulate the response of plants to environmental stress through the ABA signaling pathway [32].

SSRs are highly efficient genetic markers, which are widely used in molecular breeding research and gene mapping. The traditional SSR marker development methods take a long time, while the development of SSR markers from transcriptome data is an efficient, convenient, and low-cost method. Wang used ISSR molecular markers to explore the genetic diversity of 22 populations of *C. oliveri*, only 251 polymorphic loci were obtained [3]. In this study, MISA software was used to analyze the transcriptomes of the four tissues of *C. oliveri*, and many SSRs loci were obtained. There was no sufficient genetic background information for *C. oliveri*. In the future, EST-SSR molecular markers of *C. oliveri* can be developed to study the genetic diversity and genetic structure of *C. oliveri*, and more scientific conservation strategies can be formulated.

LncRNAs are also an important set of regulatory factors, which can interact with miRNA networks to regulate genes expression before transcription; in addition, they can be combined with enhancers, promoters and chromatin modification complexes after transcription to regulate genes expression [33]. In this study, many lncRNAs were obtained from the four tissues of *C. oliveri*, and these lncRNAs were expected to play an important role in regulating the physiological responses of *C. oliveri*.

### 3.4. Tissue-Specific Expressed Genes

Different tissues of plants play different roles in the process of environmental adaptation due to their highly specialized functions. In order to fully obtain the transcriptome information of *C. oliveri* and understand the differences between the tissues, this study conducted a transcriptomic study on the male cone, root, stem, and leaf of *C. oliveri*. GO and KEGG function enrichment analysis show the differences between the tissues.

11,650 (root), 3587 (leaf), 11,348 (male cone) and 5671 (stem) tissue-specific expressed genes were found in the four tissues. GO enrichment results show that the tissue-specific expressed genes of male cone were mainly enriched in categories related to phosphorylation, such as “phosphate-containing compound metabolic process (GO: 0006796)”, “phosphorus metabolic process (GO: 0006793)”, “phosphorylation (GO: 0016310)” and “protein phosphorylation (GO: 0006468)”. Protein phosphorylation is a kind of protein post-translational modification, which plays a vital role in abiotic stress signaling pathway [34]. The stem-specific expressed genes were mainly enriched in regulatory terms. The leaf-specific expressed genes were involved in three Go terms in response to biological stress, which may indicate that leaf have a special mechanism independent of the other three tissues when facing biotic factors. In addition, the tissue-specific expressed genes of the male cone in the GO enrichment had more types than the other three tissues, which indicated that the male cone required more physiological activities to maintain its growth and development as a reproductive organ. The KEGG enrichment analysis of all tissue-specific expressed unigenes also showed the differences between tissues. The male cone-specific expressed unigenes were mainly enriched in biosynthetic pathways, while the leaf-specific expressed unigenes were assigned to pathways related to amino acid metabolism and photosynthesis.

### 3.5. Gene Families

Dehydrins are a member of late embryogenesis abundant (LEA) proteins families. These proteins contained different conserved motifs in amino acid sequences, which were named as Y-, S- and K-motifs or A-and E-motifs specifically in gymnosperms [12]. In this study, only K- and S-type motifs were found in the dehydrins of *C. oliveri*, but did not find Y-, A- and E-type motifs. Among them, 16 dehydrins belonged to the K_n_ type, and the number of K-motif repeats was 2 to 4; 11 belonged to the SK_n_ type and contained one S-segment composed of 6 to 8 serine residues. Studies have shown that K-fragments can form an amphipathic a-helix structure, which can establish hydrophobic interactions with other proteins to stabilize the cell membrane [35,36]. Multiple K-segments can be gathered to form an intramolecular binding structure, which enhances the amphipathic characteristics of dehydrin [37]. Drira found that the K-fragment in *dhn5* from wheat plays a vital role in protecting lactate dehydrogenase and β-glycosidase [38].

Many research reports indicated that the accumulation of dehydrins was positively related to the cold and drought tolerance of plants [39,40]. The data of in vitro dehydrins research showed that dehydrins may participate in the process of plant growth and the response to dehydration stress [41]. In this study, 67 unigenes annotated as dehydrin were found in the four tissues of *C. oliveri*. It can be inferred that these unigenes play an important role in the environmental adaptability of *C. oliveri*. In addition, SK_n_ and K_n_-type dehydrin were found to highly express in root, stem, and leaf, while SK_n_-type dehydrins were mainly expressed in male cone, which may indicate that the S-motif played an important role in male cone.

Heat shock protein was a specific stress protein produced by organisms under environmental stress. The rich expression of HSP can significantly improve plant tolerance to environmental stress [42]. Small molecule heat shock protein (sHSP) was different from other heat shock protein families in that sHSP itself cannot refold unnatural proteins. They can bind to unnatural proteins through hydrophobic interaction and prevent aggregation of unnatural proteins, and then refold through other ATP-dependent molecular chaperones [43,44]. In this study, 22 unigenes annotated as small molecule heat shock proteins were identified in the four tissues of *C. oliveri*. Compared with HSP70 and HSP90, most of the sHSP were expressed at a lower level in the four tissues of *C. oliveri*.

Studies have found that the HSP70 family was the most conserved in the heat shock protein family. A variety of HSP70 had been identified in organelles such as chloroplast, endoplasmic reticulum and mitochondria in higher plants [45]. In this study, it was found that the most unigenes were annotated as HSP70 in the four tissues of *C. oliveri*. HSP70 had an important function in preventing aggregation and assisting in the refolding of unnatural proteins. Some family members of HSP70 were expressed constitutively and were often called Hsc70 (Heat shock cognate 70 kDa protein). The members of the Hsc70 family were mainly involved in assisting the folding of newly synthesized peptides and the translocation of precursor proteins, while other members of the HSP70 family were only expressed when plants were affected [46]. There were 14 unigenes annotated as Hsc70 in the four tissues of *C. oliveri*, and i2_HQ_CO3Flo_c76111/f10p0/2334 was the most highly expressed among all HSP70 family members. Unlike other molecular chaperones, most of the known substrates of HSP90 were signal transduction proteins, such as signal kinases and steroid hormone receptors [47]. HSP90 played an important role in morphological evolution and adversity adaptation of *A. thaliana* [48]. In this study, only 11 unigenes annotated as HSP90 were found in the four tissues of *C. oliveri*, but the expression of these unigenes in the four tissues was significantly higher than that of HSP70 and sHSP families.

The CYP450 superfamily is one of the largest gene families in plants and is involved in various biosynthesis pathways [49]. In this study, 91 unigenes annotated as cytochrome P450 were identified in the four tissues of *C. oliveri*. All the sequences of the four tissues can be divided into 8 clans: CYP71, CYP72, CYP86, CYP97, CYP85, CYP51, CYP74 and CYP727. Among them, the CYP71 clan had the most family members (12). The CYP71 family contains more than 50% of plant CYPs and was the largest family cluster among CYP450s [49]. Therefore, its functions were very diverse. This study also found many family members of the CYP71 clan in *C. oliveri*, and the expression of these family members in the root was significantly higher than the other three tissues, which indicated that the family clusters play an important role in the root. In addition, 7 CYP750 and 5 gymnosperm-specific members of CYP76 were found in the four tissues [50]. Studies have shown that gymnosperms CYP76AA25 and CYP750B1 can catalyze the hydroxylation of sabinene in *Thuja plicata* to produce trans-sabin-3-ol [51].

### 3.6. Characterization of the Unigenes in Plant Hormone Signal Transduction

This study found that a total of 1299 unigenes from the four tissues of *C. oliveri* were assigned to the plant hormone signal transduction (ko04075) pathway. These genes were mainly involved in the signal transduction of auxin, cytokinin, gibberellin, abscisic acid, ethylene, brassinosteroid, jasmonic acid and salicylic acid.

Abscisic acid signal transduction is an important tool for plants to produce strong stress responses to environmental stimuli. When plants face abiotic stresses such as drought and cold, they will utilize abscisic acid to assess the impact of stress and change the signal transmission pathway of abscisic acid to adjust the corresponding physiological processes [52,53]. In the signal transduction process, abscisic acid firstly binds to the receptor PYR/PYL/RCAR proteins, causing the conformation of receptors to change, and then binds to protein phosphatase PP2C. Subsequently, ternary complexes composed of ABA–PYR/PYL/RCAR–PP2C enable the activation of SnRK2s, resulting in the phosphorylation of downstream substrates, such as bZIP transcription factors and membrane channel proteins [54]. This study found that the PYL protein and ABA response binding factor (ABF) had high expression levels in the four tissues, but the expression level in the root was significantly higher than the other three tissues. This may suggest that ABA signal transduction plays an important role in the root.

Jasmonic acid (JA), a cyclopentanone plant hormone, is widely distributed in various tissues of plants, and plays an important role in physiological responses to environmental stress and in developmental processes [55]. The JAZ protein family is an important negative regulator in the jasmonic acid signaling pathway, which could bind and inhibit MYC2 transcription factor [56]. Under natural conditions, all four tissues of *C. oliveri* expressed JAZ protein, but the expression level in root was significantly higher than the other three tissues.

Salicylic acid (SA) is a phenolic derivative, widely found in higher plants. The researchers found that exogenous SA treatment could improve plant resistance to drought, low temperature and other adversity stresses [57]. This study found that a total of 117 unigenes in the four tissues of *C. oliveri* were involved in endogenous SA signaling, of which 31 were annotated as NPR1 protein (regulatory protein NPR1), and 71 were annotated as transcription factor TGA. NPR plays a positive regulatory role in the salicylic acid signaling pathway. This protein can enter the nucleus to induce PR gene to express; and TGA transcription factor is a direct connection point in this process [58]. This study found that NPR1 protein, TGA transcription factor and PR1 (pathogenesis-related protein 1) were highly expressed in the root of *C. oliveri*. The FPKM of PR1 in the root reached 1028.54, while the PFKM of leaf, male cone and stem were only 197.39, 48.39 and 14.26, respectively. This indicated that the root of *C. oliveri* may face more severe environmental stress than the male cone, leaf, and stem in natural state.

### 3.7. Characterization of the Unigenes in Circadian Rhythm-Plant

In plants, circadian rhythm regulation forms the basic adaptation of plants to fluctuating environments [59]. Plant circadian clock is mainly composed of three parts, namely input pathway, core oscillator, and output pathway. The input pathway is mainly responsible for the transmission of environmental timing information (temperature and light); while the core oscillator mainly integrates environmental signals to generate new rhythmic oscillation signals; the output pathway amplifies the new rhythmic oscillation signals and transmits them to downstream genes, causing other physiological and biochemical reactions [27]. Although different plants have different biological clock genes, the genes in core oscillator are relatively conservative. The plant core oscillator includes a core circuit, a day circuit and a night circuit. The core circuit includes the transcription factor CCA1, the pseudo-response regulator TOC1 and LHY; the daytime circuit is composed of CCA1/LHY, PRR9/PRR7/PRR5 and the night circuit is composed of TOC1, GI and ZTL [60].

This study found that 495 unigenes from the four tissues of *C. oliveri* were assigned to circadian rhythm-plant pathway (ko04712), 34 and 12 of which were annotated as LHY, TOC1, respectively. In addition, there were 8, 80, 35, and 20 unigenes annotated as PRR5, PRR7, GI, and ZTL, respectively. Studies have shown that the circadian central oscillator can regulate the expression of many genes that respond to environmental stress, making them express rhythmicity. Hamer et al. found that the transcription factor LHY regulated the response of plants to low temperature stress by controlling the expression of *CBF* gene [61]. In addition, GI can participate in the regulation of *early responsive to dehydration* (*erd10 erd7*), *cor78*, *cor15a* and other drought and cold stress response genes, and ABA or drought response genes were positively regulated by PRR7 [62]. The expression levels of these regulatory genes were different in the four tissues of *C. oliveri*. The expression levels of LHY, PRR7 and ZTL in stem and leaf were significantly higher than that in root and male cone; while PRR5 and GI were mainly expressed in root, which indicated that the regulation of circadian rhythms was different between different tissues.

## 4. Materials and Methods

### 4.1. Plant Materials and RNA Extraction

The plant materials for this experiment were collected from Xiushui Meishan (28°47′0″ N, 114°39′19″ E, altitude: 330 m), Jiangxi Province, China. The climate in Xiushui County belongs to the subtropical monsoon climate. It is cold in winter with frost and snow. Summer is hot and drought often occurs. Xiushui has maintained the highest temperature record in China except Xinjiang (National Meteorological Information Center, China Meteorological Administration). A well-grown male tree was selected in Meishan to collect four tissues samples. All samples were stored in RNAfixer (BioTeke, Shanghai, China) and stored at −20 °C until RNA extraction.

Total RNA of each tissue was extracted using the Rneasy Plus Mini kit (Qiagen, Valencia, CA, USA) according to the manufacturer’s instructions. Using 1% agarose gels to monitor RNA degradation and contamination. The purity and integrity of each sample were evaluated using Nanodrop 2000 spectrophotometer (Thermo Scientific, Wilmington, DE, USA) and Agilent Bioanalyzer 2100 system (Agilent Technologies, Santa Clara, CA, USA). Using the Qubit 2.0 fluorometer (Invitrogen, Life Technologies, Carlsbad, CA, USA) to accurately quantify the RNA concentration of all samples.

### 4.2. Illumina Library Preparation, Sequencing and de Novo Assembly

The sequencing Library was generated using the NEBNext Ultra RNA Library Prep Kit (NEB, Ipswich, MA, USA) according to the manufacturer’s instructions. Sequencing was performed on the Illumina NovaSeq platform (Illumina, San Diego, CA, USA), generating paired-end (PE) reads with lengths of 150 bp. Use in-house Perl script to filter raw reads, mainly to remove adapter reads, reads with more than 10% ambiguous bases “N”, and low-quality reads (Qphred ≤ 20 base with more than 50%). Trinity v. 2.4.0 was used to assemble clean reads of four tissues, with parameters set as min_kmer_cov: 4 and other default parameters [63].

The Illumina RNA-Seq transcriptome raw data were deposited in the SRA of NCBI as follows: root: SRR12058212; stem: SRR12058211; leaf: SRR12058210; male cone: SRR12058209.

### 4.3. PacBio Library Preparation, Sequencing and Preprocessing

Total RNA for each of the four tissues was constructed library separately according to the PacBio Isoform Sequencing (Iso-Seq) experimental protocol. PacBio libraries were sequenced on the PacBio Sequel platform (Pacific Biosciences, Menlo Park, CA, USA). To obtain Polymerase reads, raw reads were used to remove data containing 0 or 2 DNA molecules as templates. Subreads were obtained by removing the connector and the data with a length less than 50 bp. The subreads.bAM file is processed by the Circular Consensus Sequence algorithm to obtain the CCS Sequence. The above data processing is carried out in SMRTlink v. 6.0 software (http://www.pacb.com/products-and-services/analytical-sofware/smrt-analysis/), and the parameters are set as: -minLength 50, -maxLength 15000, -minPasses 2, -min_seq_len 200, -minPredictedAccuracy 0.8, -minZScore -9999, -maxDropFraction 0.8, -min_seq_len 200, -minReadScore 0.65, -no_polish TRUE.

Arrow was used to calibrate the consensus sequence using the nFL sequences [64]. And the parameters were set as: Hq_quiver_min_accuracy 0.99, bin_BY_Primer false, bin_size_KB 1, Qv_TRIM_5p 100, Qv_TRIM_3p 30. To further improving the sequencing accuracy, validate the polished consensus sequence with second-generation data using LoRDEC V0.7 software and the parameters are set as follows: -K 23, -S 3 [65].

Using CD-HIT v. 4.6.8 to cluster the corrected transcript sequences according to the 95% similarity between the sequence, and the parameters are set as: -c 0.95, -T 6, -G 0, -aL 0.00, -aS 0.99, -AS 30 [66]. Select the core conserved gene set of terrestrial plants, namely embryophyta_odb9 (Creation date: 13 February 2016, number of species: 30, number of BUSCOs: 1440), and use BUSCO v. 3.0.2 to evaluate the completeness of the full-length transcriptome sequences [67].

The PacBio Iso-Seq FL transcriptome data were deposited in the Sequence Read Archive (SRA) of NCBI as follows: root: SRR12058216; stem: SRR12058215; leaf: SRR12058214; male cone: SRR12058213.

### 4.4. Functional Annotation of Transcripts

The obtained non-redundant transcript sequences were functionally annotated using the following databases: NCBI non-redundant protein (NR) database, the EuKaryotic Orthologous Groups (KOG) database, the Swiss-Prot database, the Kyoto Encyclopedia of Genes and Genomes (KEGG) database, NCBI nucleotide sequences (NT) database, the Gene Ontology (GO) database and the Protein Family (Pfam) database. The first four databases annotations were performed using DIAMOND v. 0.8.36 with an E-value threshold of 1.0 × 10^−5^ [68]. Using ncbi-blast-2.7.1+ with an E-value threshold of 1.0 × 10^−5^ and Hmmscan of the HMMER 3.1 package (http://hmmer.org/) for NCBI Nt database annotation and Pfam database annotation [69]. Blast2GO (http://www.blast2go.com) and a script were used for GO annotations [70].

### 4.5. Prediction of CDSs, TFs, LncRNAs

Predictive analysis of CDS (Coding sequence) is performed using ANGEL v. 2.4 software [71]. The software includes no error and error tolerant modes (the default is the fault tolerant mode). The parameter setting is: --min_angel_aa_length 50, and the remaining options are the default. iTAK V1.7A software was used to predict the plant transcription factors in the four tissues of *C. oliveri* [72]. The parameters were set as follows: -F 3F. The unigenes were applied to the CNCI v. 2 [73], CPC v. 0.9 [74], PfamScan v. 1.6 [75] and PLEK v. 1.2 [76] to predict the coding potential. for lncRNA prediction through screening coding potential. Unigenes that are longer than 200 nt and do not code protein were selected as lncRNA candidates.

### 4.6. Simple Sequence Repeat (SSR) Detection

SSR, also known as microsatellite, is a tract of repetitive DNA in which certain DNA motifs (ranging in length from 2–13 base pairs) are repeated, typically 5–50 times. Unigenes from Iso-seq were selected for SSR analysis using MISA v. l.0 [77] with default parameters. The minimum repeat time for core repeat motifs was set as following: 10 for mononucleotide, 6 for dinucleotides and 5 for trinucleotides, tetranucleotides, pentanucleotides and hexanucleotides.

### 4.7. Gene Expression Quantification and DEG analysis

The CD-HIT software was used to dereduce the corrected consensus sequence by 95% similarity, and the obtained transcripts were used as the reference sequence of the gene. Using bowtie2 v. 2.3.4 [78] with the end-to-end, sensitive mode and other default parameters to align the clean reads of each tissue obtained by Illumina sequencing to the reference sequence. RSEM software was used to count the readcount value of each gene in each tissue, and then using FPKM (expected number of fragments per kilobase of transcript sequence per Millions base pairs sequenced) to determine the expression level of each unigene. DEGseq v. 1.12.0 software [79] was used for differential expression analysis of tissue genes. Unigenes with *q* value <0.005 and |log2 (fold change)| > 1 were considered to be the DEGs.

### 4.8. Enrichment Analysis of Tissue-Specific Expressed Genes

The Bingo plug-in of Cytoscape v. 3.7.2 software [80] was used for GO enrichment analysis of tissue-specific expressed genes. The software uses a hypergeometric test to identify GO functional categories that were overexpressed in tissue-specific expressed genes. Using the BH method to correct the p-value, and only GO terms with a corrected *p*-value < 0.05 were significantly enriched. GO enrichment analysis of tissue-specific expressed genes in tissues was performed using GO seq v. 1.10.0 [81]. The enrichment analysis of KEGG pathway was performed using KOBAS v. 3.0 [82]. The p-value correction method and the threshold value were consistent with the above.

### 4.9. Gene Family Analysis

Based on functional annotations from publicly available databases (Nr, Swiss-Prot, Pfam, and KOG), the environmental stress-related gene families were identified in *C. oliveri*. The number of unigenes annotated for known abiotic factor-related gene families (DHN, HSP, CYP450) were compared among the four tissues of *C. oliveri*.

Using MEME Suite [83] to predict potential functional motifs in the dehydrin family, and set the parameters as: motif -time 1000 -maxsize 600,000 -mod anr -nmotifs 10 -minw 6 -maxw 20. Based on the detected conservative motifs, using MAST v5.1.1 to classify dehydrin family with default parameters [84]. MAFFT 7 was used to perform multi-sequence alignment with default parameters [85]. Using MEGA X to build a neighbor-joining (NJ) evolutionary tree with the Poisson model [86]. Repeat 1000 times with the bootstrap method to check the branch support rate of the phylogenetic tree reconstructed by NJ.

## 5. Conclusions

In conclusion, this is the first study of transcriptomes in the four tissues of *C. oliveri.* We obtained 63,831 (root), 58,108 (stem), 33,013 (leaf) and 62,436 (male cone) full-length unigenes and the genes function and genes structure information based on the functional annotation. Furthermore, the gene families and pathways related to abiotic factors were characterized, including DHN, HSP, CYP450 families, plant hormone signal transduction and circadian rhythm-plant pathway. GO and KEGG enrichment analysis of tissue-specific expressed genes revealed the special roles in response to environmental stress and adaptability in the different four tissues. This study not only provides a practical guide for the transcriptomic analysis of species lacking genomic information but will also facilitate further studies on functional genomics, adaptive evolution, and phylogeny and lay a foundation for the development of conservation strategies for this endangered conifer.

## Figures and Tables

**Table 1:** Statistics of annotations of the full-length transcripts from four tissues of *C. oliveri* with seven databases.

Database	Male Cone	Stem	Leaf	Root
Nr	57,787	51,624	28,649	48,379
Swiss-Prot	50,366	44,725	24,361	39,525
KEGG	57,094	50,652	27,952	46,851
KOG	38,768	34,613	18,671	31,056
GO	40,828	35,387	19,395	29,940
Nt	40,691	32,627	18,436	29,751
Pfam	40,828	35,387	19,395	29,940
At least one database	58,601	52,990	29,411	51,054
All databases	23,857	18,801	10,302	14,577

**Table 2:** The number of genes of the third-level Gene Ontology (GO) terms from “response to stimulus” in each tissue.

GO_ID	GO_Term	Male Cone	Leaf	Root	Stem
GO: 0006950	Response to stress	218	124	207	190
GO: 0009733	Response to auxin stimulus	9	8	15	5
GO: 0009415	Response to water	28	53	43	63
GO: 0006979	Response to oxidative stress	68	39	105	61
GO: 0009725	Response to hormone stimulus	78	24	39	68

**Table 3:** The number of differentially expressed genes of tissues compared in pairs.

Degs Set Name	All Degs Number	Up-Regulated Degs Number	Down-Regulated Degs Number
Male cone vs. leaf	10,343	5371	4972
Male cone vs. root	12,021	5779	6242
male cone vs. stem	9465	4897	4568
Leaf vs. root	9704	4262	5442
Leaf vs. stem	1634	1076	558
Root vs. stem	8385	5046	3339

## Data Availability

The data that support the findings of this study are available from the corresponding author upon reasonable request.

## Supplementary Materials

Supplementary materials can be found at https://www.mdpi.com/1422-0067/22/2/787/s1. Table S1. Summary for the transcriptome data of C. oliveri using PacBio Iso-Seq; Table S2. Statistics of the unigenes sequenced using PacBio Iso-Seq; Table S3. Summary of the reads sequenced using Illumina RNA-Seq; Table S4. Statistics of the unigenes sequenced using Illumina RNA-Seq; Table S5. Identification of transcription factors (TFs) in four tissues of *C. oliveri*; Table S6. The mapping between the Illumina reads of each sample and the reference transcript sequences generated by PacBio Iso-Seq; Table S7. Gene Ontology (GO) enrichment analysis of tissue-specific expressed genes; Table S8. The Kyoto Encyclopedia of Genes and Genomes (KEGG) pathway enrichment analysis of tissue-specific expressed genes; Table S9. Summary of the dehydrin (DHN) gene family; Table S10. Summary of the heat shock protein (HSP) gene family. Table S11. Summary of the cytochrome P450 (CYP450) gene family. Table S12. Statistics of the unigenes in the plant hormone signal transduction (ko04075) pathway; Table S13. Statistics of the unigenes in the circadian rhythm-plant (ko04712) pathway; Figure S1. Overview of the data processing pipeline; Figure S2. The results of the transcriptome integrity assessment based on BUSCO using an Embryophyta (ODB9) core gene dataset. The number of Embryophyta gene sets used in this evaluation was 1440; Figure S3. The distribution of homologous species annotated in the NCBI non-redundant protein (Nr) database. (a) root; (b) male cone; (c) leaf; (d) stem; Figure S4. Gene Ontology (GO) classification analysis of unigenes; Figure S5. The Kyoto Encyclopedia of Genes and Genomes (KEGG) pathway classification statistics of the unigenes; Figure S6. The biological process category of GO enrichment analysis of tissue-specific expressed genes. (a) stem; (b) leaf; Figure S7. The "molecular function" category of GO enrichment analysis of tissue-specific expressed genes. (a) male cone; (b) root; Figure S8. The molecular function category of GO enrichment analysis of tissue-specific expressed genes. (a) stem; (b) leaf.
