# Supplementary material for: Full-Length Transcriptome Analysis of Four Different Tissues of Cephalotaxus oliveri
Source: Int J Mol Sci. 2021 Jan 14;22(2):787. doi: 10.3390/ijms22020787 (PMC7830723; doi:10.3390/ijms22020787)
Supplement: Supplementary file 1 [file ijms-22-00787-s001.zip › ijms-1046333-supplementary/ijms-1046333 supplementary materials final/ijms-1046333- Supplementary Figures and Tables S1-4,6.docx]

**Supplementary Files**

Full-Length Transcriptome Analysis of Four Different Tissues of *Cepholotaxus oliveri*

Ziqing He ^1^, Yingjuan Su ^1,2,^* and Ting Wang ^3,^*

^1^ School of Life Sciences, Sun Yat-sen University, Guangzhou 510275, China; hezqlife@163.com

^2^ Research Institute of Sun Yat-sen University in Shenzhen, Shenzhen 518057, China

^3^ College of Life Sciences, South China Agricultural University, Guangzhou 510642, China

***** Correspondence: suyj@mail.sysu.edu.cn (Y.S.); tingwang@scau.edu.cn (T.W.); Tel.: +86-020-84111939 (Y.S.); +86-020-85280185 (T.W.)

**Table S1.** Summary for the transcriptome data of *C. oliveri* using PacBio Iso-Seq.

| **Sample** | **Reads Bases (G)** | **Reads Number** | **Mean Length** | **N50** | |
| --- | --- | --- | --- | --- | --- |
| **Polymerase Reads** | | | | | |
| CO3_Root | 11.08 | 312,574 | 35,452 | 59,348 | |
| CO3_Stem | 19.79 | 353,127 | 56,040 | 86,138 | |
| CO3_Leaf | 11.74 | 213,338 | 55,046 | 87,997 | |
| CO3_Flo | 21.25 | 560,605 | 37,898 | 62,604 | |
| **Subreads** | | | | | |
| CO3_Root | 10.45 | 8,444,499 | 1,238 | 1488 | |
| CO3_Stem | 19.11 | 9,113,656 | 2,097 | 2875 | |
| CO3_Leaf | 11.12 | 7,873,312 | 1,412 | 1912 | |
| CO3_Flo | 20.54 | 9,465,158 | 2,171 | 2677 | |
| **Sample** | **Reads Number** | **Min Length** | **Max Length** | **Mean Length** | **N50** |
| CCS | | | | | |
| CO3_Root | 264,227 | 51 | 13,945 | 1724 | 2058 |
| CO3_Stem | 303,178 | 50 | 14,892 | 2868 | 3304 |
| CO3_Leaf | 189,764 | 50 | 14,794 | 2208 | 2996 |
| CO3_Flo | 462,122 | 50 | 14,677 | 2693 | 3051 |
| FLNC | | | | | |
| CO3_Root | 213,507 | 200 | 13,668 | 1520 | 1804 |
| CO3_Stem | 268,740 | 200 | 14,429 | 2716 | 3179 |
| CO3_Leaf | 150,247 | 200 | 14,605 | 2040 | 2844 |
| CO3_Flo | 401,815 | 200 | 14,568 | 2506 | 2869 |
| **Polished Consensus** | | | | | |
| CO3_Root | 143,177 | 189 | 13,668 | 1563 | 1941 |
| CO3_Stem | 158,855 | 171 | 14,447 | 2719 | 3240 |
| CO3_Leaf | 96,459 | 163 | 14,804 | 2046 | 2879 |
| CO3_Flo | 209,217 | 150 | 14,568 | 2490 | 2937 |

**Table S2.** Statistics of the unigenes sequenced using PacBio Iso-Seq.

| **Sample** | **<500 bp** | **500–1 kbp** | **1–2 kbp** | **2–3 kbp** | **>3 kbp** | **Total** | **Mean Length** | **N50** | **N90** |
| --- | --- | --- | --- | --- | --- | --- | --- | --- | --- |
| Root | 4130 | 12,893 | 25,060 | 10,140 | 11,608 | 63,831 | 1980 | 2523 | 1037 |
| Stem | 815 | 3465 | 10,527 | 16,522 | 26,779 | 58,108 | 2973 | 3480 | 1926 |
| Male cone | 1697 | 4975 | 11,031 | 6554 | 8756 | 33,013 | 2357 | 3181 | 1251 |
| Leaf | 953 | 4166 | 15,969 | 17,797 | 23,551 | 62,436 | 2736 | 3267 | 1712 |

**Table S3.** Summary of the reads sequenced using Illumina RNA-Seq.

| **Sample** | **Raw Reads** | **Clean Reads** | **Clean Bases (G)** | **Error (%)** | **Q20 (%)** | **Q30 (%)** | **GC Content (%)** |
| --- | --- | --- | --- | --- | --- | --- | --- |
| Root | 53,465,116 | 52,585,224 (98.35%) | 7.89 G | 0.03 | 97.03 | 91.99 | 43.95 |
| Stem | 51,141,786 | 50,074,864 (97.91%) | 7.51 G | 0.03 | 97.01 | 91.94 | 44.27 |
| Leaf | 49,657,744 | 48,633,650 (97.94%) | 7.30 G | 0.03 | 97.08 | 92.13 | 44.88 |
| Male cone | 56,215,624 | 53,481,146 (95.14%) | 8.02 G | 0.02 | 98.26 | 94.85 | 44.30 |

**Table S4.** Statistics of the unigenes sequenced using Illumina RNA-Seq.

| **Sample** | **200–500 bp** | **500–1 kbp** | **1–2 kbp** | **>2 kbp** | **Total** | **Mean Length** | **N50** | **N90** |
| --- | --- | --- | --- | --- | --- | --- | --- | --- |
| Root | 13,122 | 19,070 | 24,198 | 15,966 | 72,356 | 1408 | 1905 | 693 |
| Stem | 6574 | 10,529 | 14,437 | 11,832 | 43,372 | 1593 | 2150 | 798 |
| Male cone | 6669 | 10,751 | 14,368 | 11,056 | 42,844 | 1527 | 2061 | 769 |
| Leaf | 5441 | 8778 | 12,707 | 9520 | 36,446 | 1550 | 2056 | 790 |

**Table S5: is in a separate Excel document.**

**Table S6.** The mapping between the Illumina reads of each sample and the reference transcript sequences generated by PacBio Iso-Seq.

| **Sample** | **Total Reads** | **Total Mapped** |
| --- | --- | --- |
| Male Cone | 53,481,146 | 48,227,272 (90.18%) |
| Leaf | 48,633,650 | 43,922,320 (90.31%) |
| Root | 52,585,224 | 33,697,206 (64.08%) |
| Stem | 50,074,864 | 44,716,176 (89.30%) |


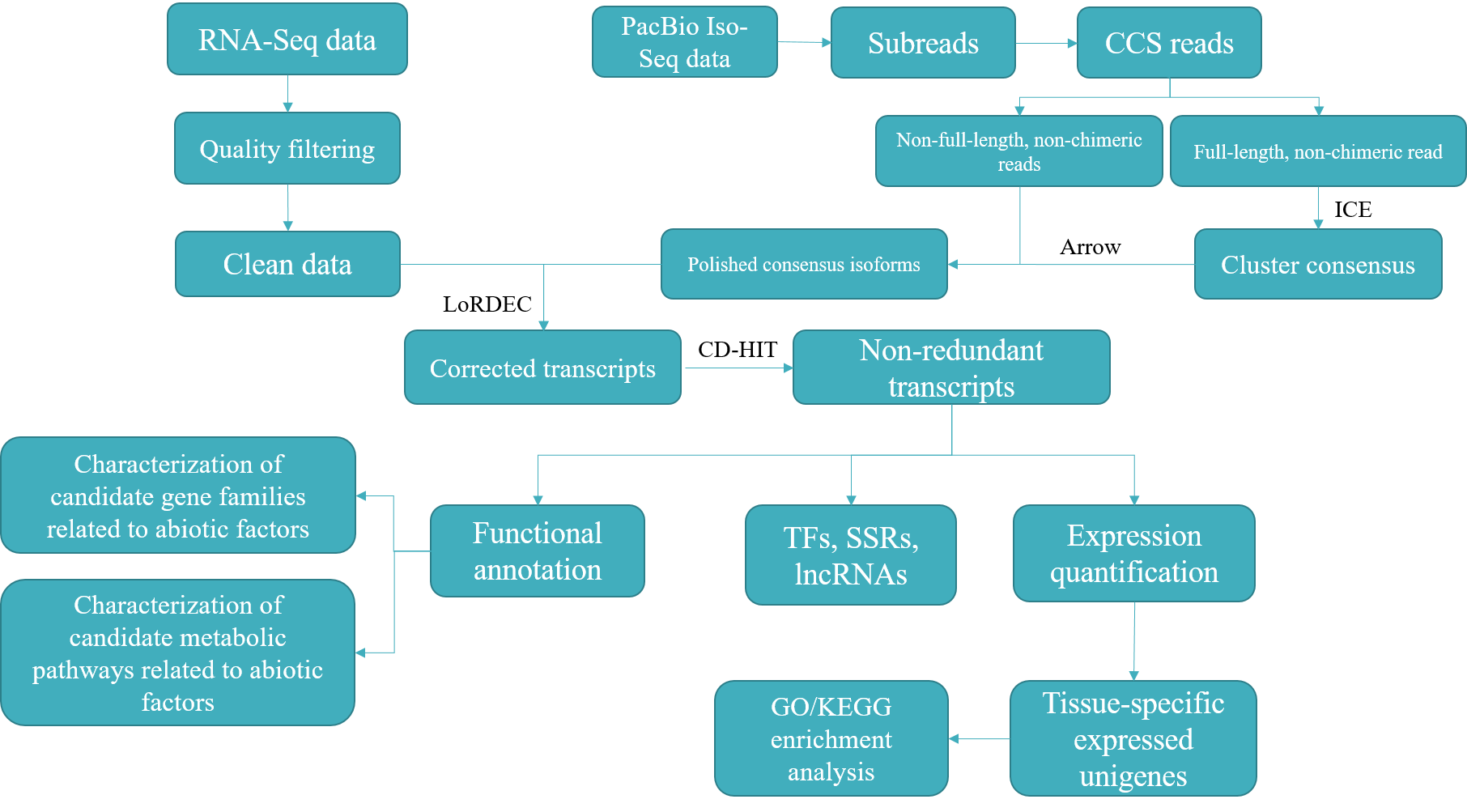


**Figure S1.** Overview of the data processing pipeline.


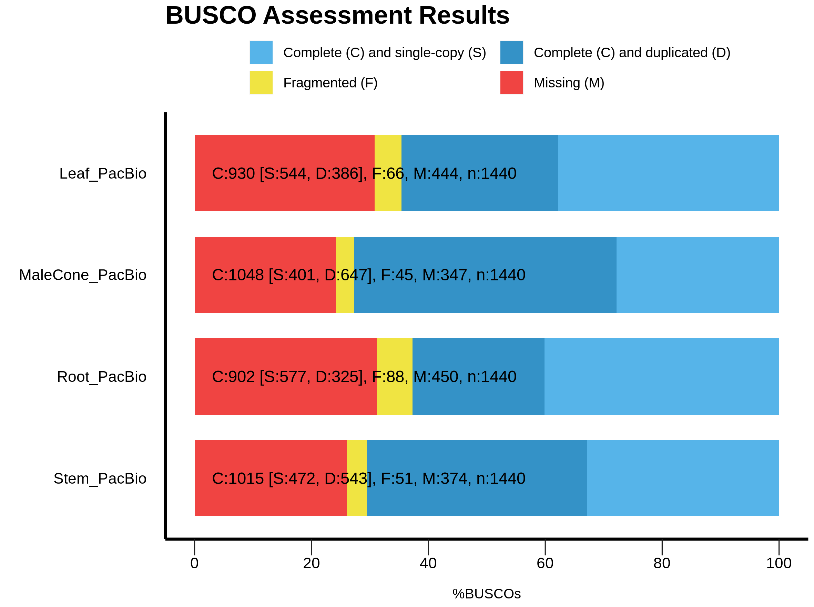


**Figure S2.** The results of the transcriptome integrity assessment based on BUSCO using an Embryophyta (ODB9) core gene dataset. The number of Embryophyta gene sets used in this evaluation was 1440.


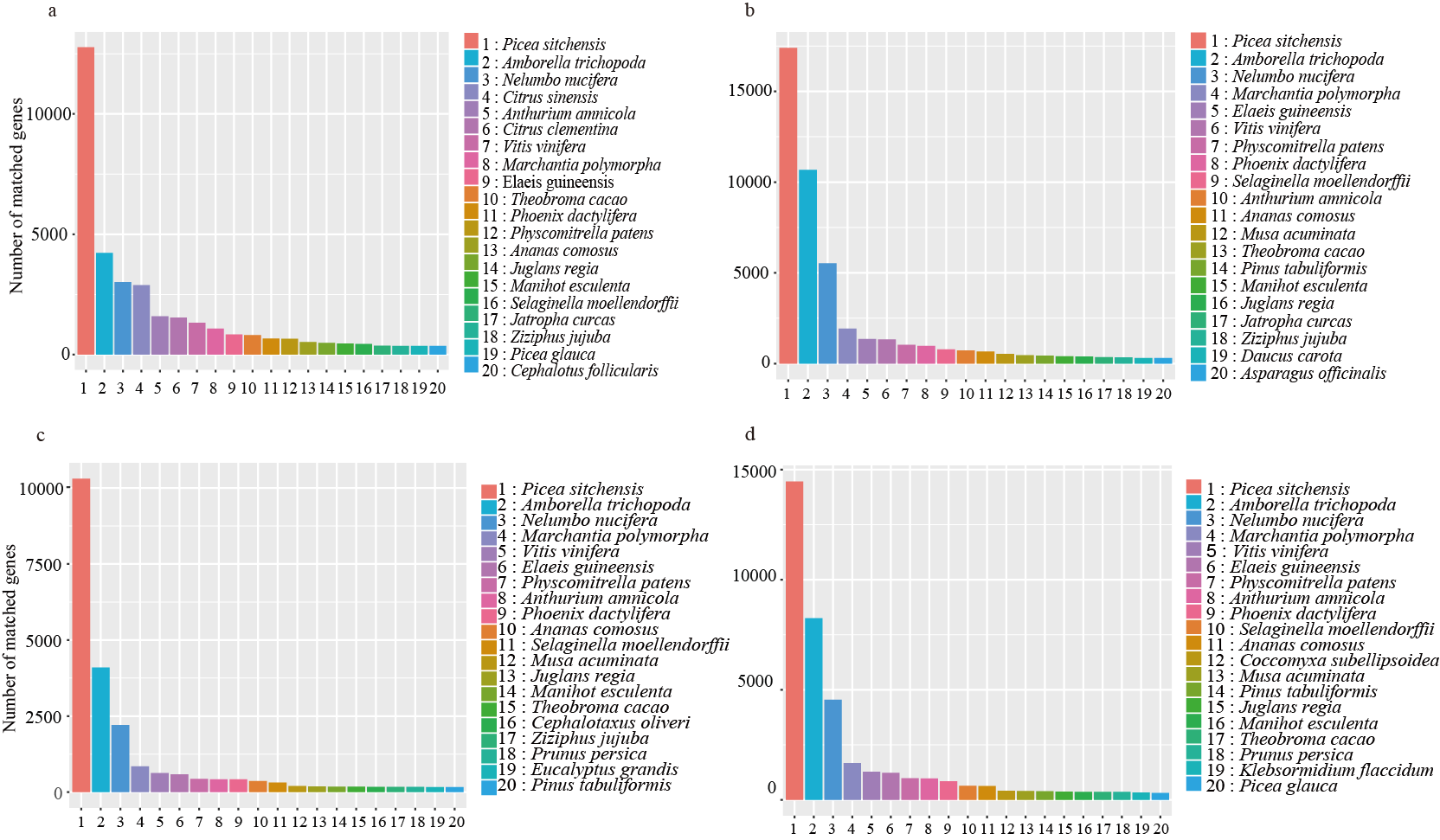


**Figure S3.** The distribution of homologous species annotated in the NCBI non-redundant protein (Nr) database. (**a**) root; (**b**) male cone; (**c**) leaf; (**d**) stem.


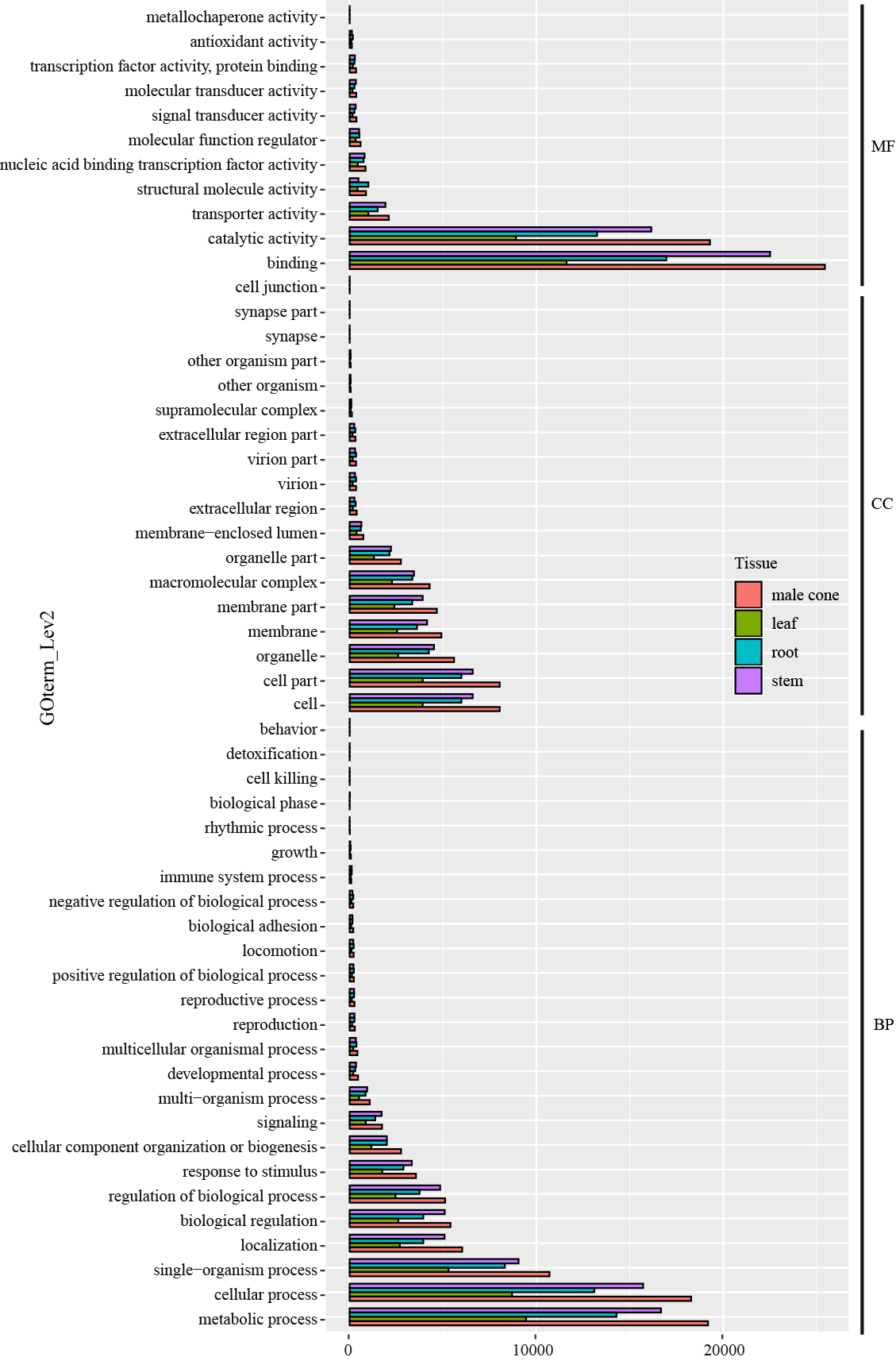


**Figure S4.** Gene Ontology (GO) classification analysis of unigenes.


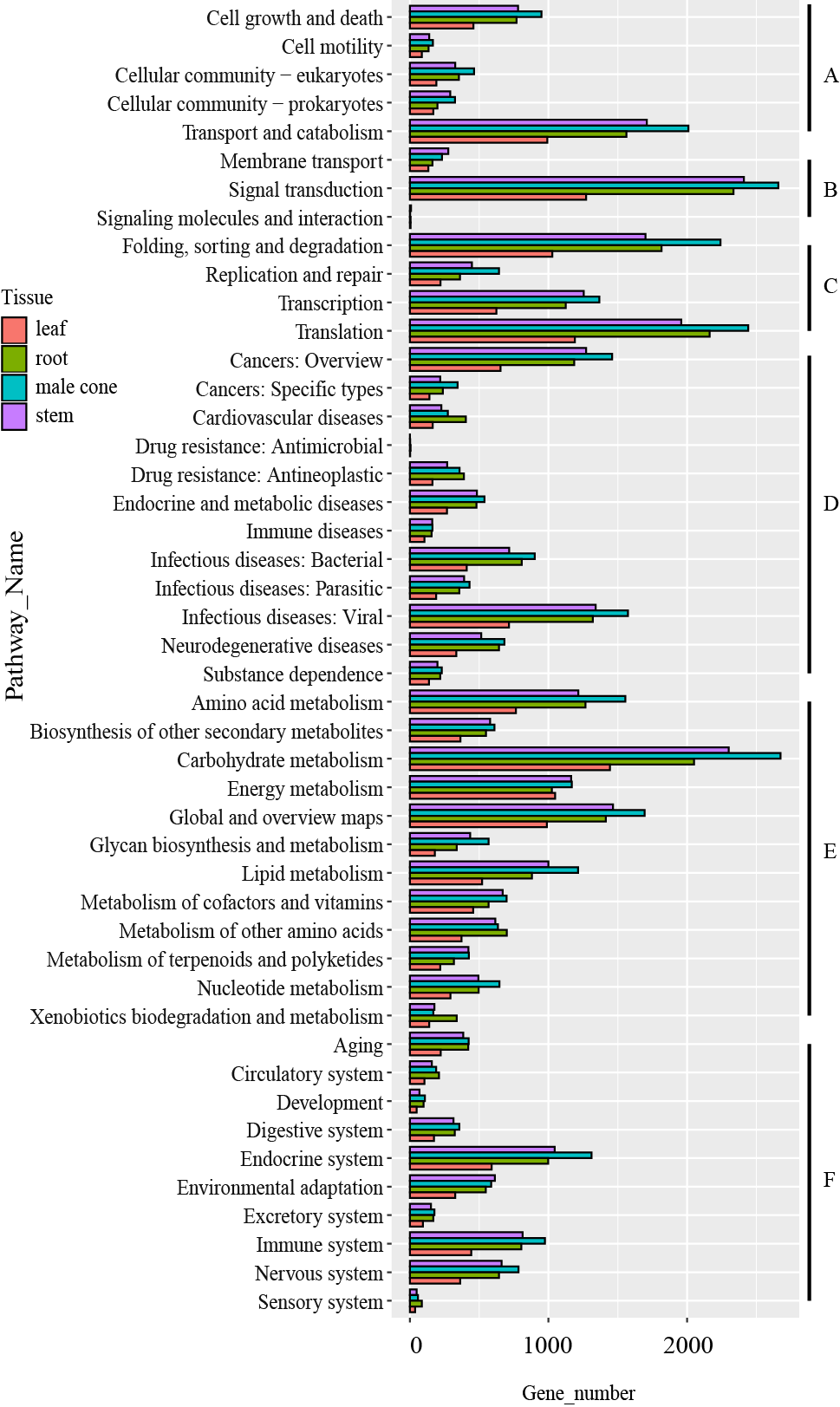


**Figure S5.** The Kyoto Encyclopedia of Genes and Genomes (KEGG) pathway classification statistics of the unigenes. (**A**) cellular processes; (**B**) environment information processing; (**C**) genetic information processing; (**D**) human diseases; (**E**) metabolism; (**F**) organismal systems).


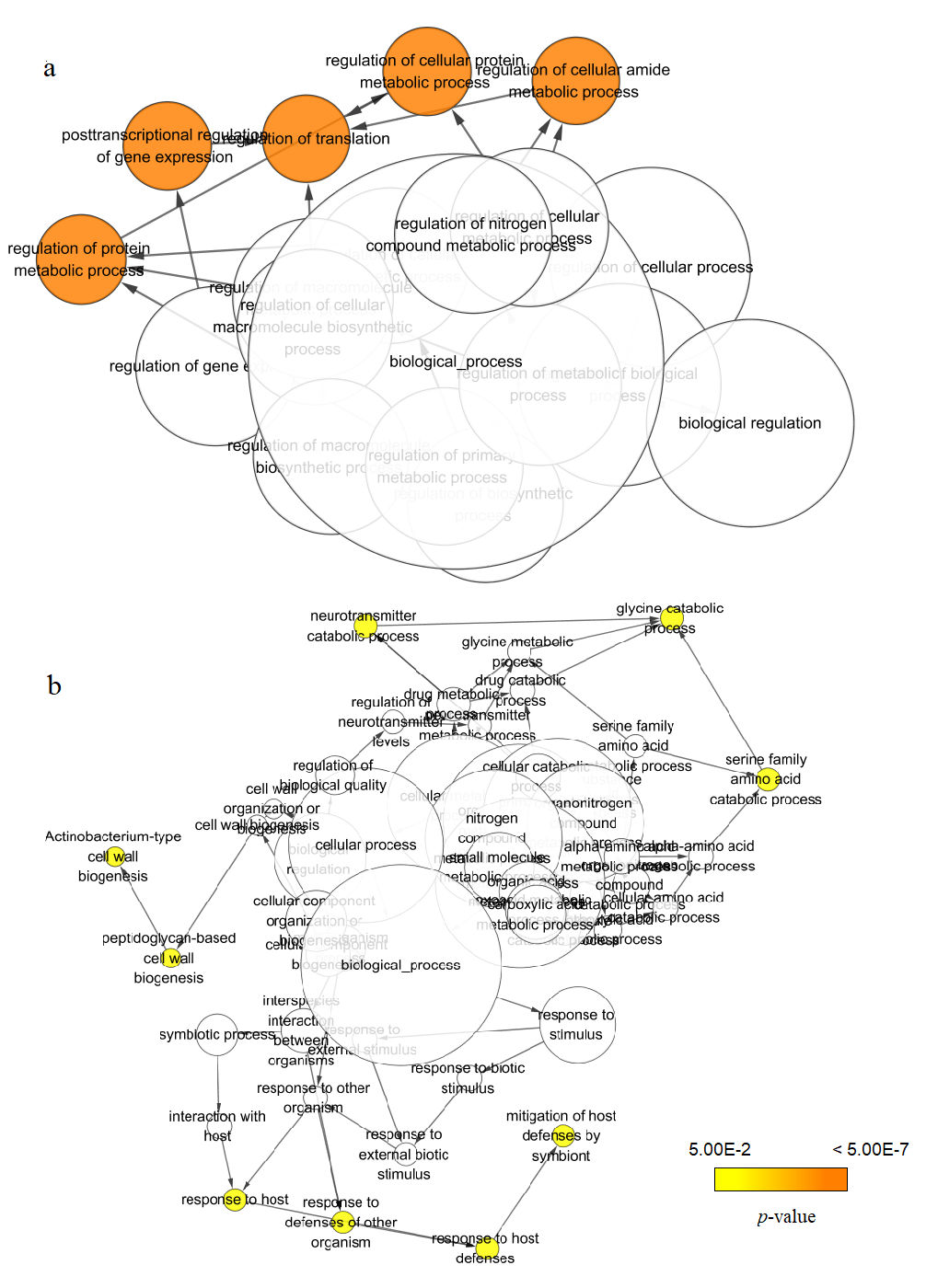


**Figure S6.** The biological process category of GO enrichment analysis of tissue-specific expressed genes. (**a**) stem; (**b**) leaf.


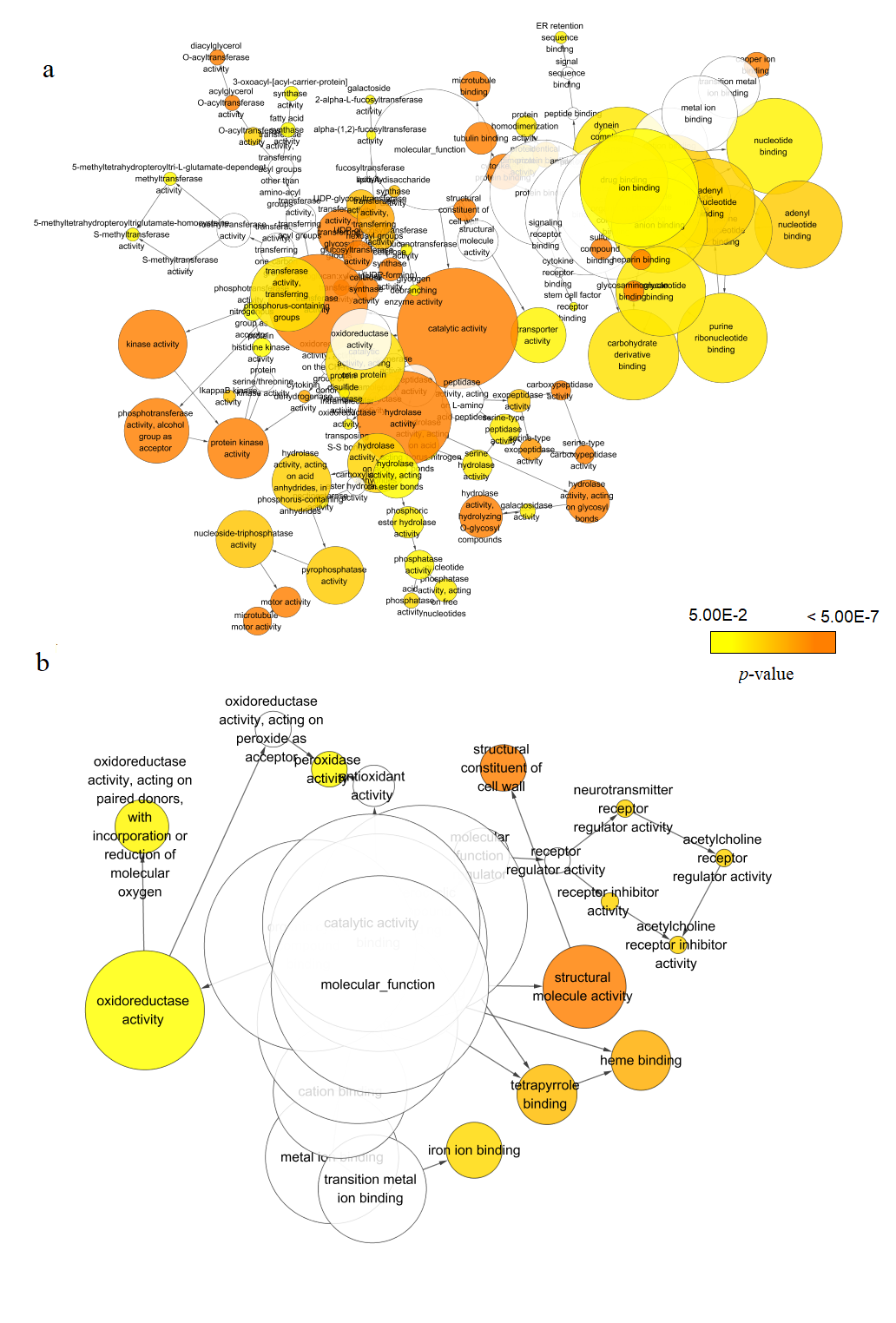


**Figure S7.** The "molecular function" category of GO enrichment analysis of tissue-specific expressed genes. (**a**) male cone; (**b**) root.


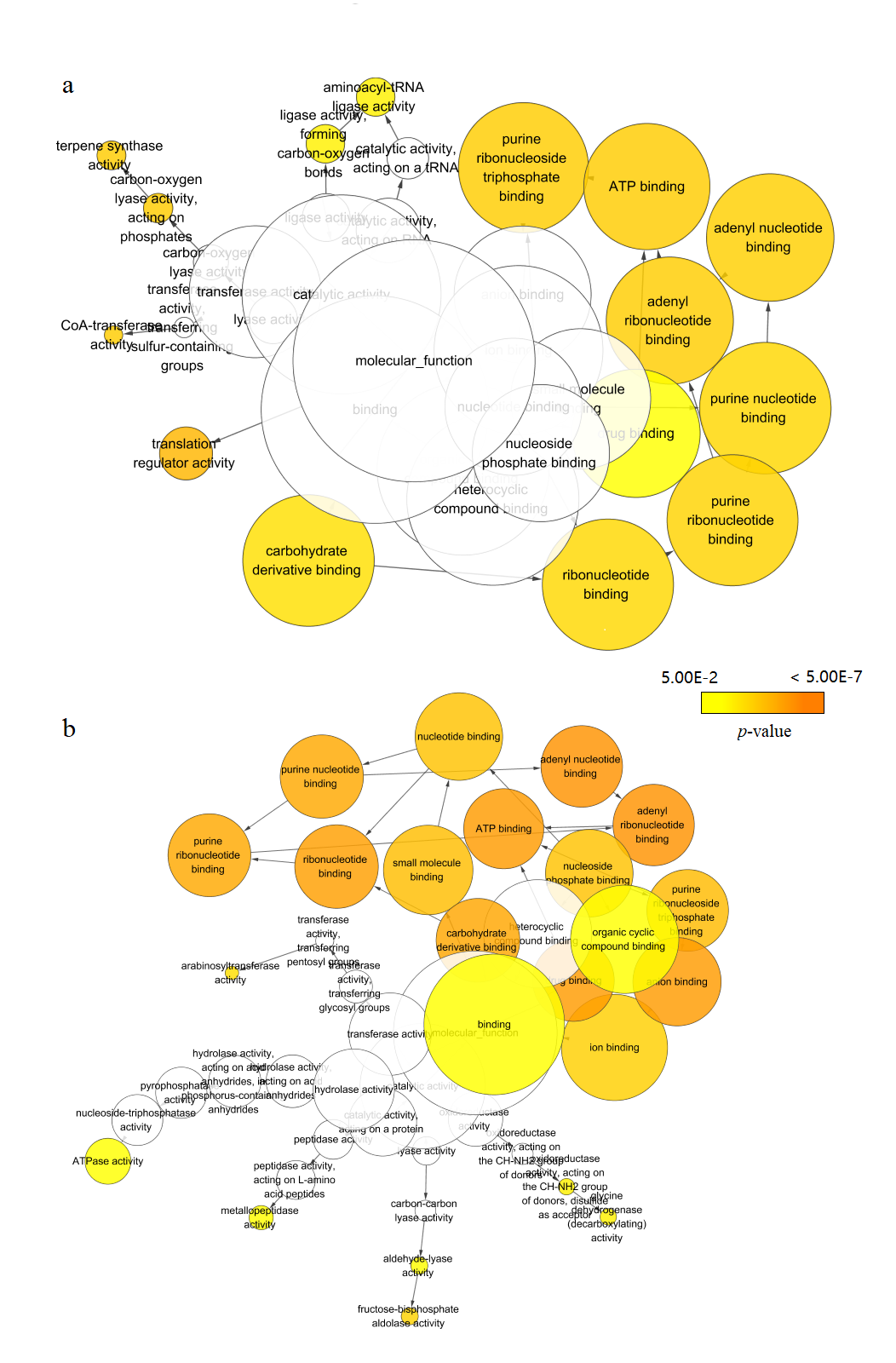


**Figure S8.** The molecular function category of GO enrichment analysis of tissue-specific expressed genes. (**a**) stem; (**b**) leaf.
